# Functional potential of gut microbiota in *Rhinolophus ferrumequinum* revealed by 16S rRNA sequencing and metabolomics

**DOI:** 10.3389/fmicb.2026.1938216

**Published:** 2026-09-23

**Authors:** Boyu Liu, Xinlei Huang, Alimire Tulake, Minghan Tian, Lei Zhou, Yujia Chu, Huimin Zhou, Tai Song, Jiang Feng

**Affiliations:** 1 College of Animal Science and Technology, Jilin Agricultural Science and Technology College, Jilin, China; 2 College of Life Science, Jilin Agricultural University, Changchun, China; 3 College of Veterinary Medicine, Jilin Agricultural University, Changchun, China; 4 Jilin Provincial Key Laboratory of Animal Resource and Ecological Security, Jilin Agricultural University, Changchun, China

**Keywords:** 16S rRNA gene sequencing, Chiroptera, fecal metabolome, functional prediction, gut microbiota, untargeted metabolomics

## Abstract

**Introduction:**

Insectivorous bats consume insects that are rich in proteins and chitin, which may impose specific requirements on microbial-mediated nutrient transformation. However, the composition and functional potential of their gut microbiota remain poorly understood.

**Methods:**

In this study, the greater horseshoe bat (*Rhinolophus ferrumequinum*), a Chiropteran species belonging to the family Rhinolophidae, was investigated to characterize fecal microbial communities and fecal metabolic profiles through the integration of 16S rRNA gene sequencing and untargeted metabolomics.

**Results:**

The fecal microbiota of *Rhinolophus ferrumequinum* exhibited diverse bacterial communities, with *Lactococcus*, *Fusobacterium*, and *Enterococcus* as dominant genera, collectively accounting for approximately 45% of the total bacterial relative abundance. Functional prediction suggested enrichment of pathways involved in lipid and amino acid metabolism, particularly tryptophan and tyrosine metabolism. Correlation analysis further identified associations between several bacterial genera, including *Acinetobacter*, *Serratia*, *Fusobacterium*, and *Mycoplasma*, and lipid-related metabolites, particularly acylcarnitines and bile acid-associated metabolites.

**Discussion:**

These findings characterize a distinct microbiota–metabolite profile in *Rhinolophus ferrumequinum* and suggest that gut microbial communities may contribute to metabolic adaptation in insectivorous bats.

## Graphical abstract

**Figure:**
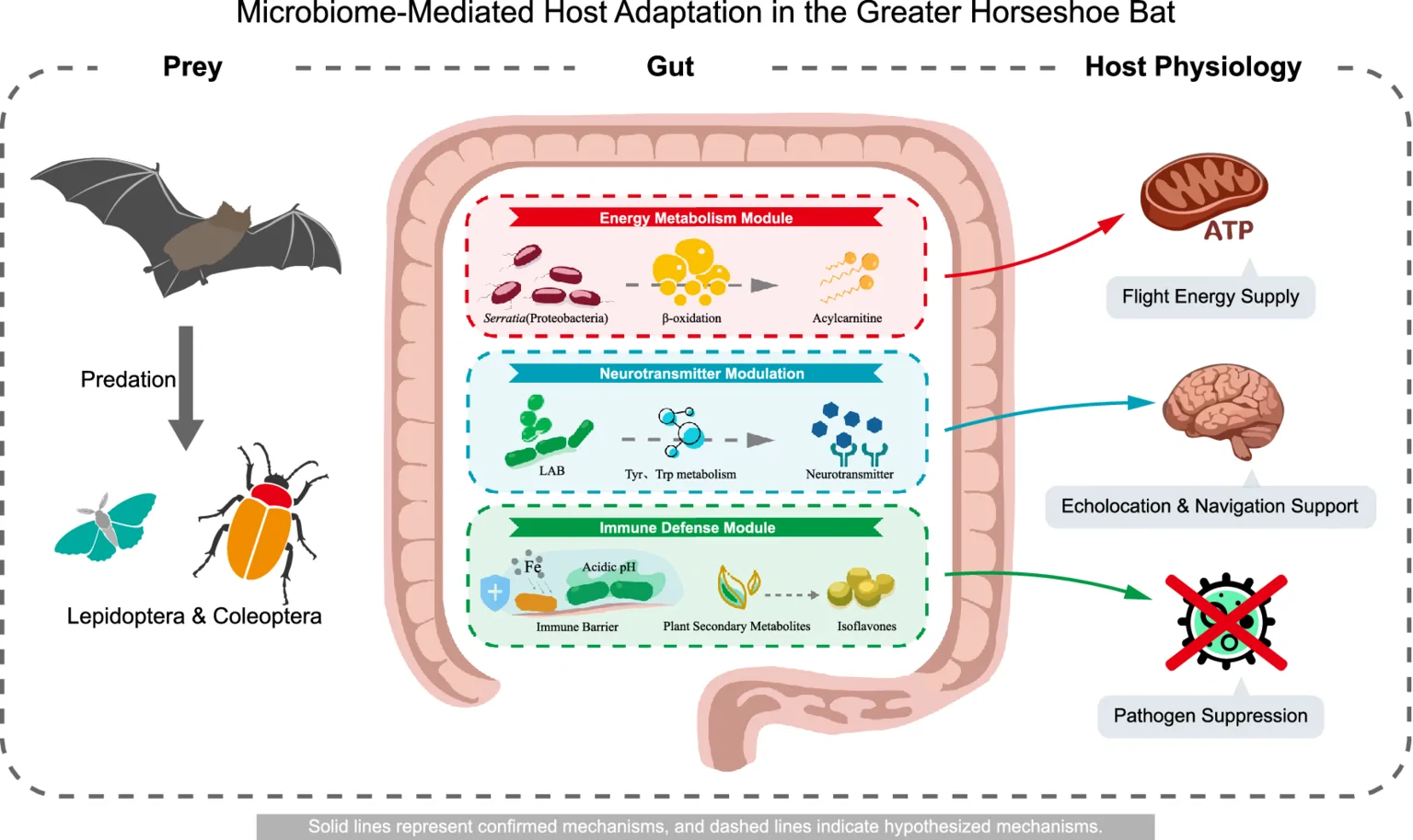

## Introduction

The order Chiroptera is the second largest order of mammals, comprising approximately 1,500 species that are widely distributed across diverse ecosystems. Bats are also the only mammals capable of sustained powered flight. During flight, their metabolic rate can increase to 15 to 30 times the resting level, resulting in exceptionally high demands for energy acquisition and oxidative metabolism (Shen et al., 2010). However, the microbial and metabolic adaptations underlying these unique ecological and physiological traits remain poorly understood.

The gut microbiota may contribute to host physiology through nutrient transformation and metabolite production, with potential implications for metabolic homeostasis and immune regulation, although its functional significance in bats remains largely unresolved (Jones et al., 2022; Melville et al., 2025b). Previous studies have suggested that gut microbial composition and predicted functional profiles may vary among bats with different dietary preferences, with differences reported in pathways related to amino acid and fatty acid metabolism (Zepeda Mendoza et al., 2018; Ingala et al., 2021). However, whether these microbial differences contribute to host adaptation remains an open question. Insectivorous bats consume insects containing abundant proteins, lipids, and chitin, and microbial communities may contribute to the transformation of these dietary components (Strobel et al., 2013). Similar relationships between specialized diets and gut microbial adaptations have been reported in other wildlife species with chitin-rich diets, highlighting the potential importance of microbiota-mediated nutrient processing in ecological adaptation (Teullet et al., 2023; Wang et al., 2026). Nevertheless, the functional characteristics of gut microbiota in insectivorous bats remain poorly understood.

Previous studies have begun to characterize the taxonomic composition of bat gut microbiota across diverse species and ecological contexts, suggesting that microbial communities may vary in association with host dietary specialization (Li et al., 2018; Dai et al., 2024; Morni et al., 2025). However, the functional significance of these microbial communities in bats remains incompletely understood, particularly regarding their potential contributions to nutrient transformation and metabolite production. Although members of Pseudomonadota and Bacillota are frequently detected as dominant taxa across bat species, these broad taxonomic patterns alone provide limited insight into the functional mechanisms underlying dietary adaptation. Moreover, integrated studies combining microbial community profiling with metabolomic analyses remain relatively scarce, especially in insectivorous bats. To address this knowledge gap, we selected *Rhinolophus ferrumequinum* as the study species because it is a widely distributed cave-dwelling insectivorous bat species, with a geographic range extending across Eurasia, including northeastern China. As a typical insectivorous bat species, *Rhinolophus ferrumequinum* provides a valuable subject for investigating host–microbiota interactions. We investigated its fecal microbiota and metabolite profiles by integrating 16S rRNA gene sequencing and untargeted metabolomics. We hypothesized that the fecal microbiota of *Rhinolophus ferrumequinum* exhibits distinct microbial and metabolic characteristics associated with its insectivorous diet and high-energy lifestyle.

## Materials and methodology

### Sample collection

In August 2023, fecal samples were collected from five adult male *Rhinolophus ferrumequinum* individuals captured in Ji’an City, Jilin Province, China (41.05°N, 125.88°E). Bats were captured using mist nets during their nocturnal foraging activity within the same sampling period. Species identification was performed based on morphological characteristics following previously published taxonomic criteria, and adult status was determined based on external morphological characteristics (Racey, 2011). All individuals were identified as males based on morphological examination. Each bat was individually placed in a sterile kraft paper bag, and freshly deposited fecal pellets were collected every 5 min over a 30-min period. After fecal collection, all bats were released at the original capture site. Fecal samples collected from the same individual were pooled and treated as one biological replicate. The pooled samples were then divided into aliquots for microbiome and metabolomic analyses. Aliquots designated for microbiome analysis were suspended in phosphate-buffered saline containing 20% glycerol to preserve microbial community integrity during long-term storage. These samples were immediately frozen in liquid nitrogen, transported to the laboratory, and stored at −80 °C until DNA extraction. Aliquots intended for metabolomic analysis were processed separately without glycerol supplementation, immediately frozen in liquid nitrogen, and stored at −80 °C until metabolite extraction.

### DNA extraction and 16S rRNA gene sequencing

Genomic DNA was extracted from fecal samples using the E.Z.N.A.^®^ Soil DNA Kit (Omega Bio-Tek, Norcross, GA, United States) according to the manufacturer’s instructions. Prior to DNA extraction, fecal samples were subjected to bead beating using 500 mg of beads and 1 mL of SLX-Mlus Buffer at 45 Hz for 250 s. The V3–V4 hypervariable region of the bacterial 16S rRNA gene was amplified using the primer pair 338F (5′-ACTCCTACGGGAGGCAGCAG-3′) and 806R (5′-GGACTACHVGGGTWTCTAAT-3′). PCR amplification was performed as previously described (Gong et al., 2021).

Raw sequencing data were processed and analyzed using the Majorbio Cloud Platform (www.majorbio.com; accessed on November 1, 2025) provided by Majorbio Bio Pharm Technology Co., Ltd. (Shanghai, China). Raw reads were quality filtered using fastp (v0.19.6), merged with FLASH (v1.2.7), and denoised using DADA2 to generate amplicon sequence variants (ASVs). The ASV table was rarefied to 30,524 sequences per sample based on the minimum sequencing depth among samples. Taxonomic annotation of ASVs was performed using the RDP Classifier against the SILVA 138 database with a confidence threshold of 0.7. Alpha diversity indices, including Shannon, Simpson, Chao1, and ACE, were calculated using mothur (v1.30) based on the rarefied ASV table. Beta diversity was assessed using Bray–Curtis dissimilarity and unweighted UniFrac distances based on the rarefied ASV table. Principal coordinate analysis (PCoA) was performed to visualize differences in microbial community composition among samples. Representative ASV sequences were used to construct a phylogenetic tree using FastTree with an approximately maximum likelihood approach. The resulting tree was midpoint rooted and used for unweighted UniFrac distance calculations. Statistical analyses and data visualization were performed using R (v3.3.1). Functional profiles of the microbial communities were predicted using PICRUSt2 (v2.2.0-b) based on ASV abundance data. The normalized ASV abundance table was subsequently converted into relative abundance and aggregated at the genus level for downstream correlation analysis. Spearman correlation analysis was performed to evaluate the associations between bacterial genera and metabolites identified by untargeted metabolomics based on normalized metabolite peak intensities. Significant correlations were defined as those with an absolute correlation coefficient (|r|) > 0.8 and *p* < 0.05.

### Untargeted liquid chromatography mass spectrometry metabolomics

Untargeted metabolomic analysis was performed using a Thermo Fisher UHPLC Q Exactive HF X system in both positive and negative electrospray ionization modes. Fecal samples (100 mg) were extracted with a methanol/water solution (4:1, v/v) containing an internal standard. After tissue homogenization, ultrasonic extraction, and centrifugation, the supernatant was collected for analysis. Chromatographic separation was performed using an HSS T3 column (100 mm × 2.1 mm, 1.8 μm; Waters). The mobile phases consisted of water and acetonitrile containing 0.1% formic acid and a mixture of acetonitrile, isopropanol, and water. The flow rate was 0.40 mL/min, and the column temperature was maintained at 40 °C. Mass spectra were acquired over an *m/z* range of 70 to 1,050. Quality control (QC) samples were analyzed throughout the analytical run to monitor instrument stability.

Raw data were processed using Progenesis QI for peak extraction, peak alignment, and metabolite annotation. Metabolites were identified by comparison with the Human Metabolome Database (HMDB), METLIN, and the Majorbio in house database. After filtering and imputing missing values, normalizing the total peak area, and retaining features with a relative standard deviation of less than 30% in QC samples, principal component analysis, HMDB and KEGG annotation, KEGG pathway enrichment, pathway topology analysis, and Spearman correlation analysis were performed using the Majorbio Cloud Platform (www.majorbio.com; accessed on November 1, 2025).

## Results

### Gut microbial composition of *Rhinolophus ferrumequinum* based on 16S rRNA gene sequencing

A total of five fecal samples were analyzed by 16S rRNA gene sequencing. A total of 1,886 amplicon sequence variants (ASVs) were identified, with an average of 377.2 ASVs per sample. The sequencing data were rarefied to 30,524 sequences per sample (Supplementary Table 1). The rarefaction curves based on the Sobs index approached saturation with increasing sequencing depth (Supplementary Figure 1), indicating that the sequencing depth was sufficient to capture the microbial diversity of the samples. The results of alpha diversity analysis based on Chao1, ACE, Shannon, and Simpson indices are presented in Supplementary Figures 2, 3. Principal coordinates analysis based on Bray Curtis distances at the ASV level showed that the first two principal coordinates explained 65.72% of the total variation in community composition, with PCo1 and PCo2 accounting for 37.38 and 28.34%, respectively (Figure 1). The PCoA plot based on unweighted UniFrac distance is presented in Supplementary Figure 4. The contribution of individual ASVs to the PCoA axes was assessed, and the detailed results are presented in Supplementary Tables 2, 3.

**Figure 1:**
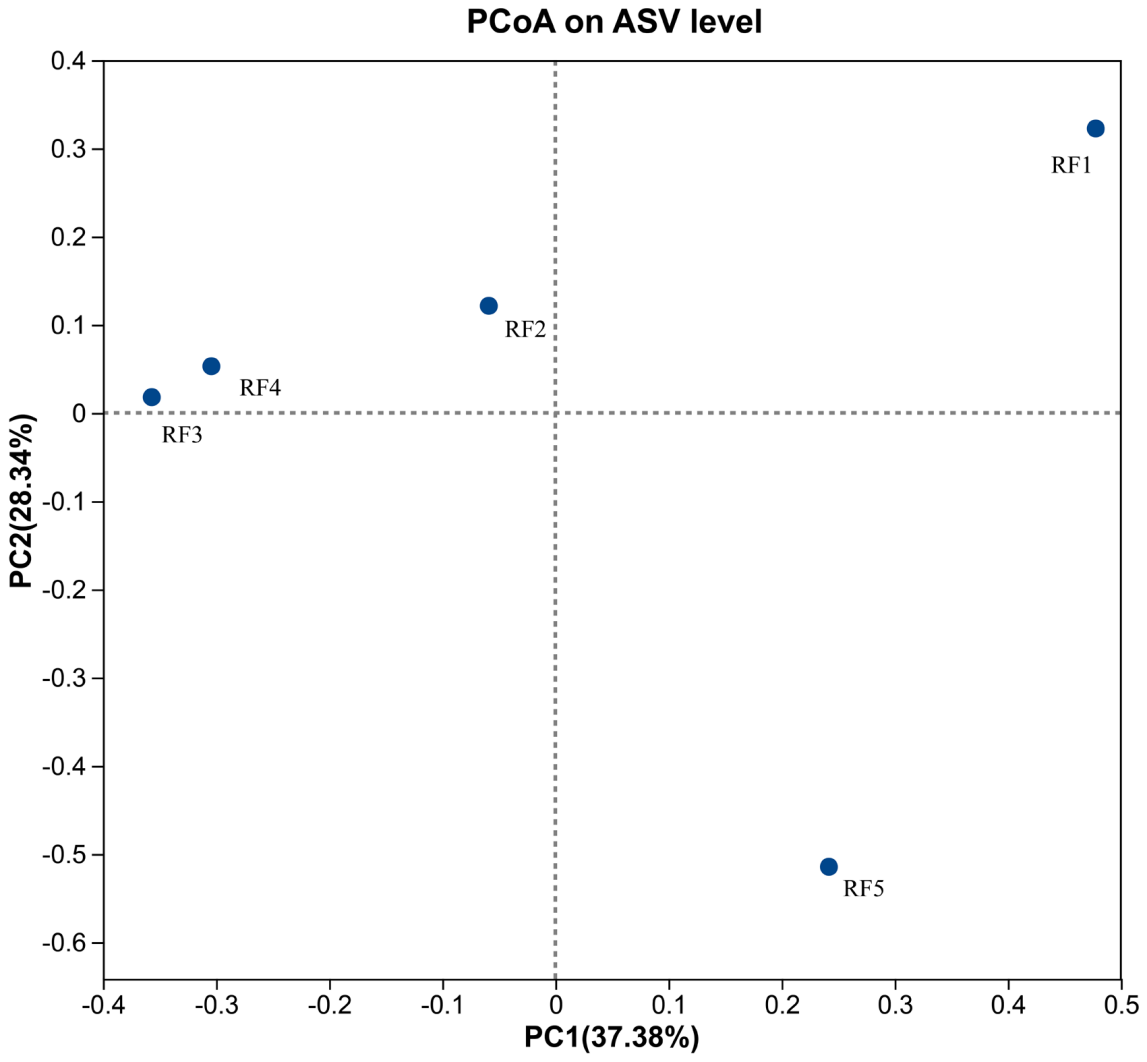
PCoA analysis of microbial communities based on ASVs. PCo1 (37.38%) and PCo2 (28.34%) represent the two principal coordinates contributing most significantly to sample differentiation. RF1 to RF5 denote five independent biological replicate samples.

Taxonomic analysis showed that the identified ASVs were assigned to 26 bacterial phyla. Bacillota (51.78%), Pseudomonadota (26.09%), and Fusobacteriota (10.60%) were the predominant phyla, followed by Bacteroidota (5.26%), Actinobacteriota (3.69%), and Synergistota (1.28%) (Figure 2A; Supplementary Table 4). The Sankey diagram showed that although individual variation was observed among samples, these dominant phyla were detected in all samples, indicating that the gut microbial community exhibited both individual variation and shared taxonomic characteristics (Figure 2C).

**Figure 2:**
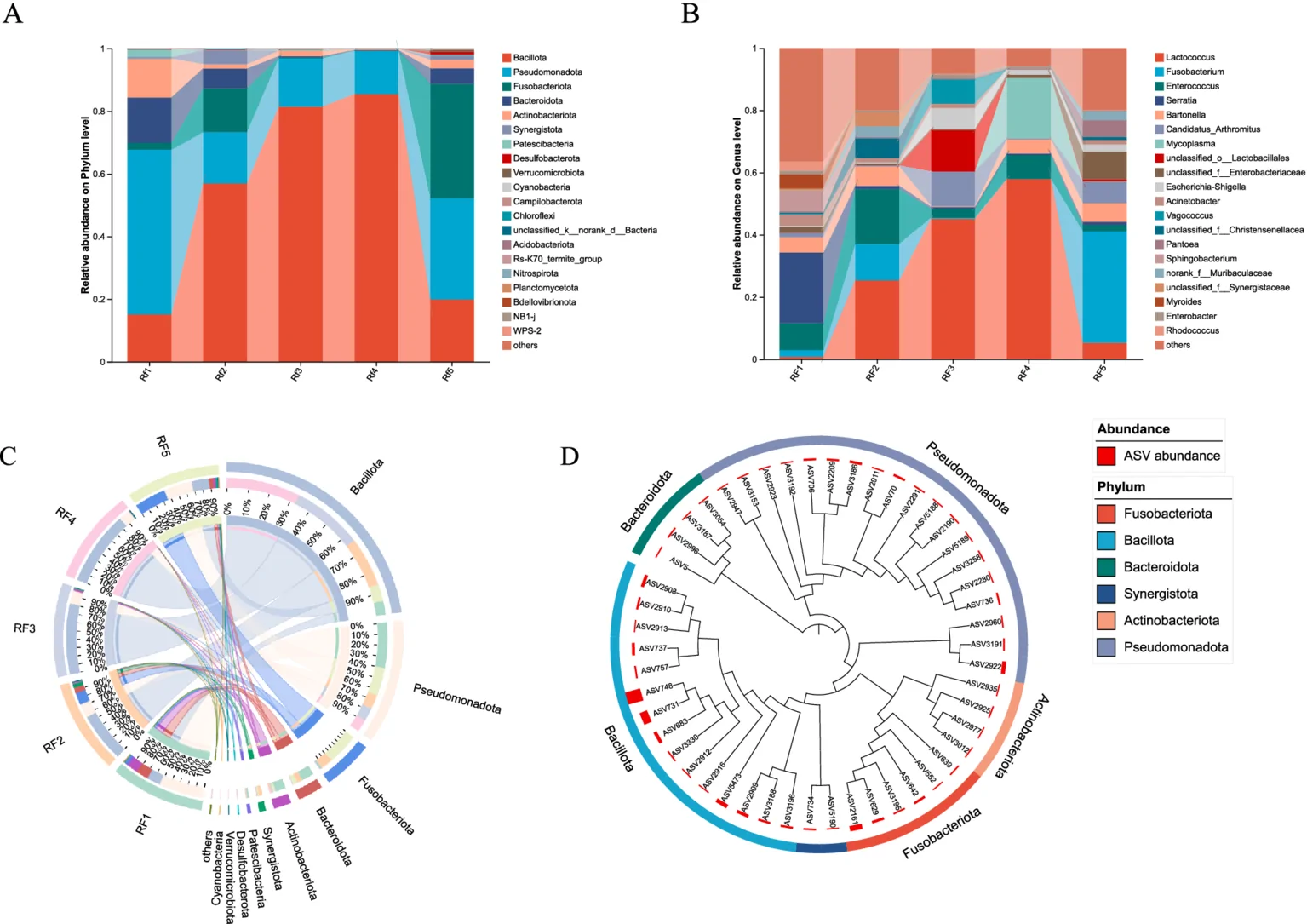
Analysis of microbial community composition and diversity. **(A)** Stacked bar chart of relative microbial community abundance at the phylum level. **(B)** Stacked bar plot of relative microbial community abundance at the genus level. **(C)** Circos chord diagram illustrating the relationship between samples (RF1–RF5) and dominant phyla. The left semicircle represents different samples, while the right semicircle denotes different phyla; the width of internal lines indicates the relative abundance of that phylum within the corresponding sample. **(D)** Phylogenetic tree of dominant ASV.

At the genus level, *Lactococcus* (26.92%) was the most abundant genus, followed by *Fusobacterium* (9.99%) and *Enterococcus* (7.77%). Other dominant genera with relative abundances exceeding 1% included *Serratia* (5.17%), *Bartonella* (4.40%), *Candidatus Arthromitus* (4.03%), *Mycoplasma* (3.90%), unclassified Lactobacillales (2.80%), unclassified Enterobacteriaceae (2.50%), and *Escherichia-Shigella* (2.30%) (Figure 2B; Supplementary Table 5). With the exception of Mycoplasma, all of these dominant taxa belonged to Bacillota, Fusobacteriota, or Pseudomonadota. A phylogenetic tree was constructed based on representative dominant ASVs to visualize the phylogenetic relationships among the dominant microbial taxa (Figure 2D).

### Functional prediction of the fecal microbiota of *Rhinolophus ferrumequinum* based on 16S rRNA gene sequencing

Functional profiles were predicted using PICRUSt2. At the broad KEGG level 1 classification, metabolic functions represented the largest functional category across all samples (Supplementary Table 6). Analysis of the predicted KEGG Orthology (KO) abundances based on PICRUSt2 showed that K01990 (ABC 2 type transport system ATP binding protein), K01992 (ABC 2 type transport system permease protein), K07024 (conserved hypothetical protein with unknown function), and K06147 (ATP binding cassette transporter, bacterial B family) were among the most abundant KOs. In addition, relatively high predicted abundances were also observed for K02529 (transcriptional regulator), K01223 (glycoside hydrolase family protein), K02004 (iron complex outer membrane receptor protein), K02003 (iron complex periplasmic substrate binding protein), and K02015 (iron complex transport system ATP binding protein) (Figure 3A).

**Figure 3:**
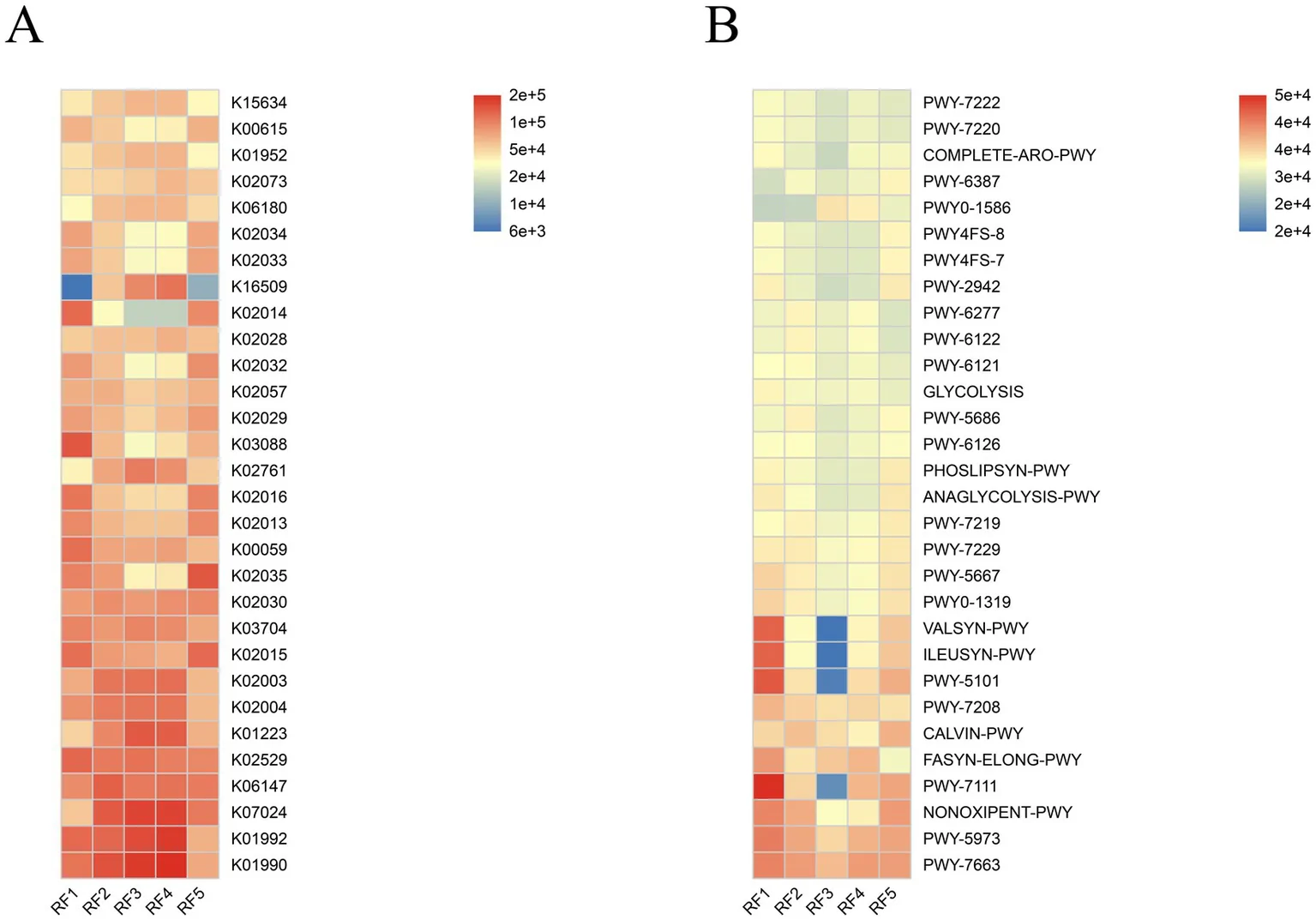
Functional prediction analysis of microbial communities. **(A)** Functional gene (KEGG Orthology, KO) abundance heatmap based on the KEGG database, with the vertical axis representing different KO functional codes and the horizontal axis representing different samples within the randomised field group. **(B)** Metabolic pathway abundance heatmap based on the MetaCyc database, with the vertical axis representing metabolic pathway names.

Metabolic pathway prediction based on PICRUSt2 and the MetaCyc database showed that multiple core metabolic pathways were predicted to be present in all samples. These included glycolysis (GLYCOLYSIS), the superpathway of fatty acid biosynthesis (PWY 7663), and the phospholipid biosynthesis pathway (PHOSLIPSYN PWY), although their predicted relative abundances varied among samples (Figure 3B). Pathways involved in valine biosynthesis (VALSYN PWY), isoleucine biosynthesis (ILEUSYN PWY), and fermentation, including PWY 7220, PWY 7222, and PWY 7111, showed relatively high predicted abundances in some samples, suggesting variation in the predicted metabolic profiles among samples. As these analyses were based on functional prediction from 16S rRNA gene sequencing data, the results represent the potential metabolic capacity of the microbial community rather than actual gene expression or metabolic activity.

### Fecal metabolite profiles of *Rhinolophus ferrumequinum* determined by LC-MS

A total of 2,575 metabolites were identified in *Rhinolophus ferrumequinum* fecal samples by LC–MS, including 1,458 and 1,117 metabolites detected in the positive and negative ion modes, respectively. Principal component analysis (PCA) showed that the first two principal components explained 62.30% of the total variance, with PC1 and PC2 accounting for 37.60 and 24.70%, respectively. The PCA score plot revealed a certain degree of separation among samples, indicating differences in their metabolic profiles (Figure 4).

**Figure 4:**
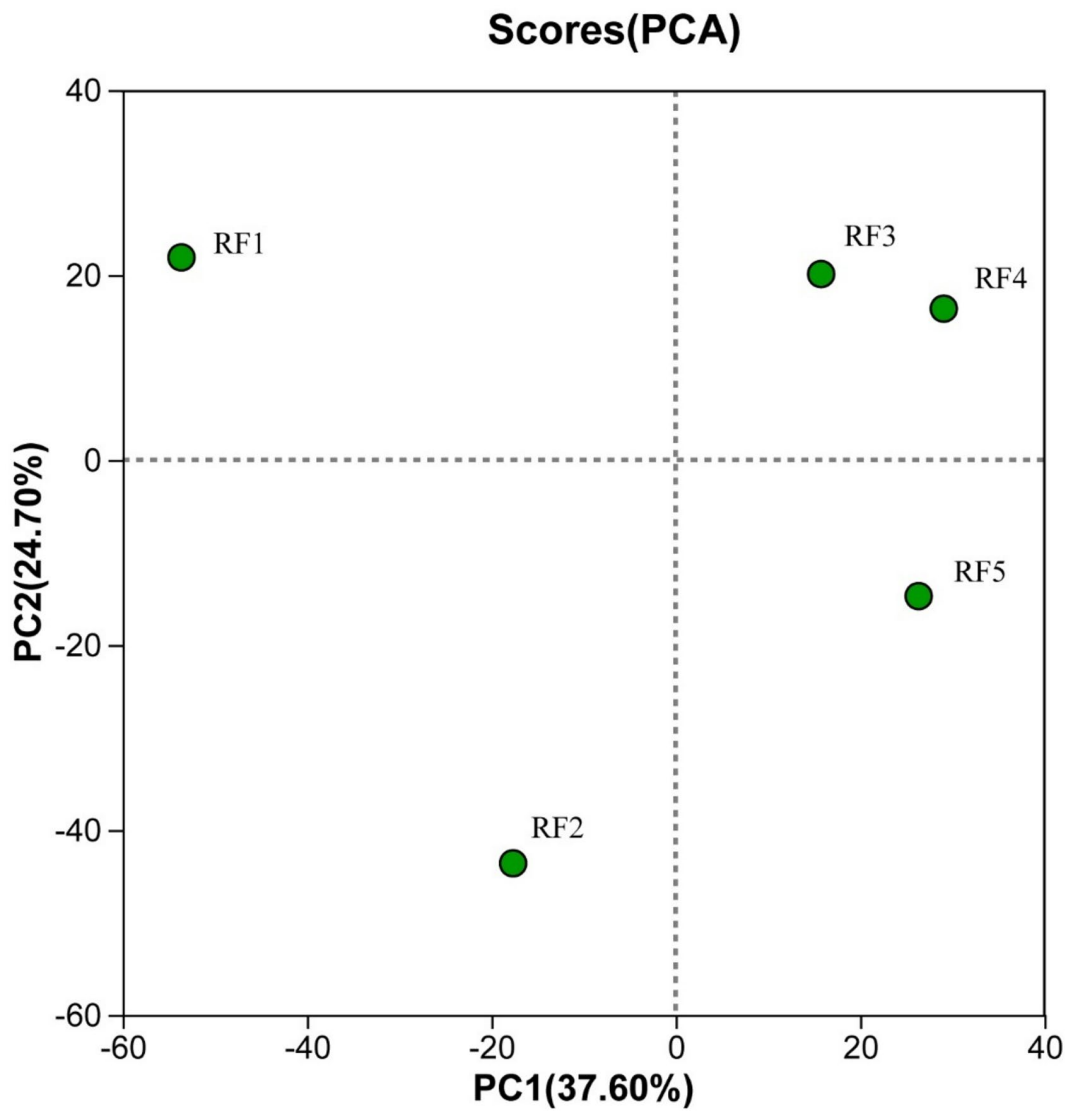
PCA analysis of sample metabolomes. PC1 (37.60%) and PC2 (24.70%) represent the two principal coordinates contributing most significantly to sample differentiation. RF1 to RF5 denote five independent biological replicate samples.

HMDB superclass annotation showed that the detected metabolites were mainly classified as lipids and lipid-like molecules (597 metabolites) and organic acids and derivatives (587 metabolites). Other major categories included organoheterocyclic compounds (398 metabolites), organic oxygen compounds (220 metabolites), benzenoids (195 metabolites), and phenylpropanoids and polyketides (127 metabolites) (Figure 5A). Within these dominant metabolite classes, several biologically relevant compounds were detected, including multiple carnitine derivatives, taurocholic acid, and lysophosphatidylcholine LysoPC (18:1) among lipid-associated metabolites, as well as amino acid-related metabolites such as L-tyrosine, glutamate, aspartate, and valine. The remaining metabolite classes are listed in Supplementary Table 7. Overall, the metabolic profiles were dominated by lipids and small organic acids, together with a considerable proportion of aromatic metabolites.

**Figure 5:**
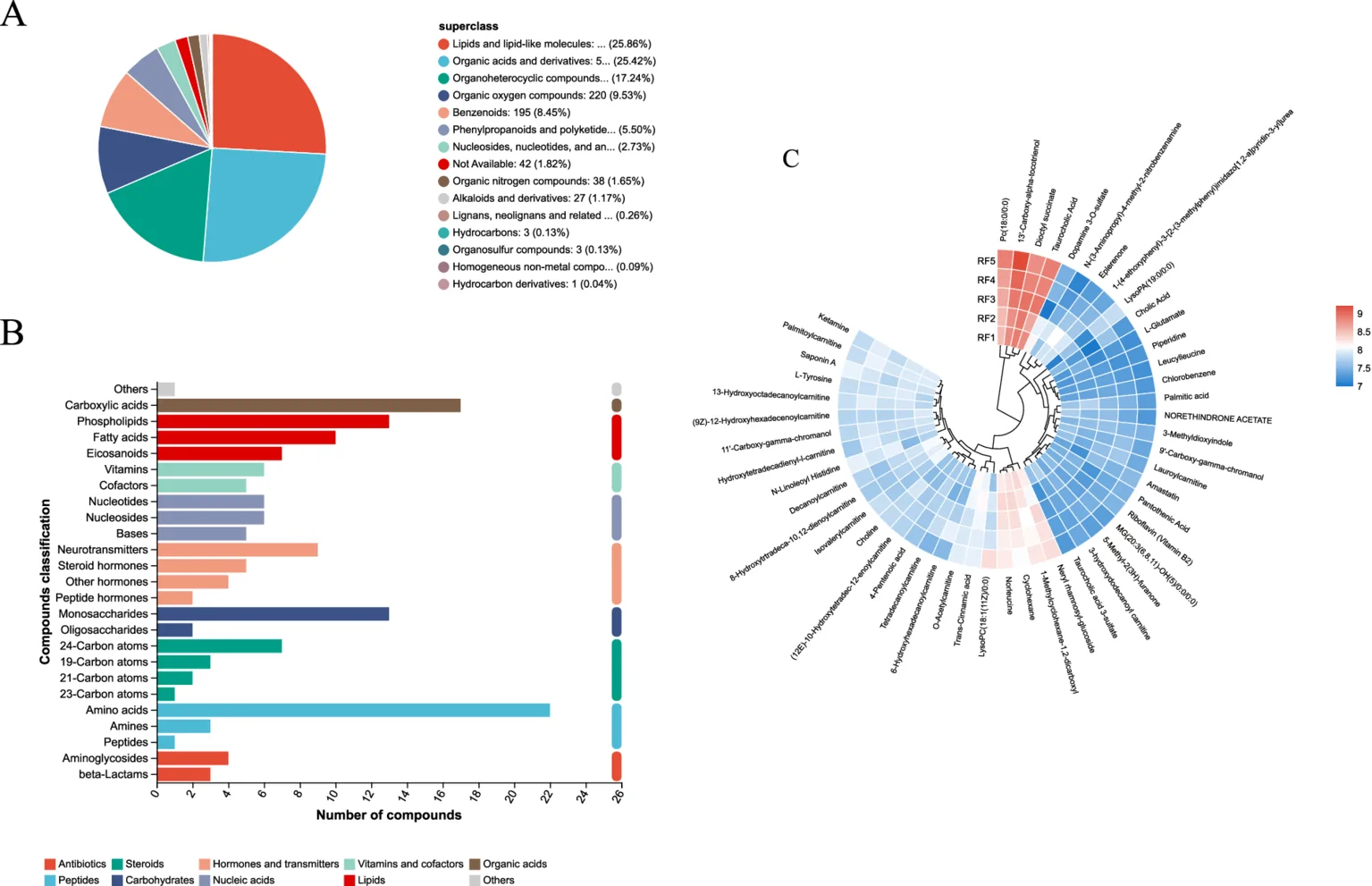
Chemical classification and KEGG functional annotation of metabolites **(A)** Annotation results at the superclass level based on the HMDB database **(B)** Compound classification annotation results based on the KEGG database **(C)** Clustering heatmap of the top 50 differentially expressed metabolites. Data underwent log10 transformation to visualise metabolite peak area intensity. The central dendrogram represents hierarchical clustering of metabolites based on abundance patterns.

KEGG compound classification further indicated that the detected metabolites were mainly distributed among lipid-related compounds (30 metabolites), peptides (26 metabolites), and hormone- and neurotransmitter-related compounds (20 metabolites), followed by nucleic acids and organic acids (17 metabolites each) (Figure 5B; Supplementary Table 8). Consistent with the HMDB-based annotation, lipid-associated metabolites and amino acid/peptide-related compounds represented major components of the fecal metabolic profiles. In addition, several metabolites annotated as neurotransmitter-related compounds were detected, including dopamine, L-Dopa, norepinephrine, epinephrine, and acetylcholine.

To characterize the major fecal metabolites of *Rhinolophus ferrumequinum*, hierarchical clustering analysis was performed based on the top 50 metabolites ranked by peak intensity after log10 transformation (Figure 5C). The clustering pattern indicated a relatively conserved metabolic profile among individuals, while several metabolites showed individual-level variation. Lipids and lipid-derived metabolites represented the predominant metabolite class, suggesting that lipid-associated compounds constitute an important component of the fecal metabolome of *Rhinolophus ferrumequinum*. Among the highly abundant metabolites, phosphatidylcholine (PC(18:0/0:0)), 13’-Carboxy-alpha-tocotrienol, and taurocholic acid exhibited relatively high signal intensities. In addition, multiple acylcarnitines, including isovalerylcarnitine, decanoylcarnitine, O-acetylcarnitine, and tetradecanoylcarnitine, were detected, indicating the presence of diverse lipid transport and fatty acid metabolism-related metabolites.

### Metabolic pathway enrichment and key pathway identification of the *Rhinolophus ferrumequinum* fecal microbiota

KEGG pathway annotation revealed that the detected metabolites were mainly assigned to pathways related to amino acid metabolism, biosynthesis of other secondary metabolites, and lipid metabolism, followed by metabolism of cofactors and vitamins and xenobiotics biodegradation and metabolism (Figure 6A; Supplementary Table 9).

**Figure 6:**
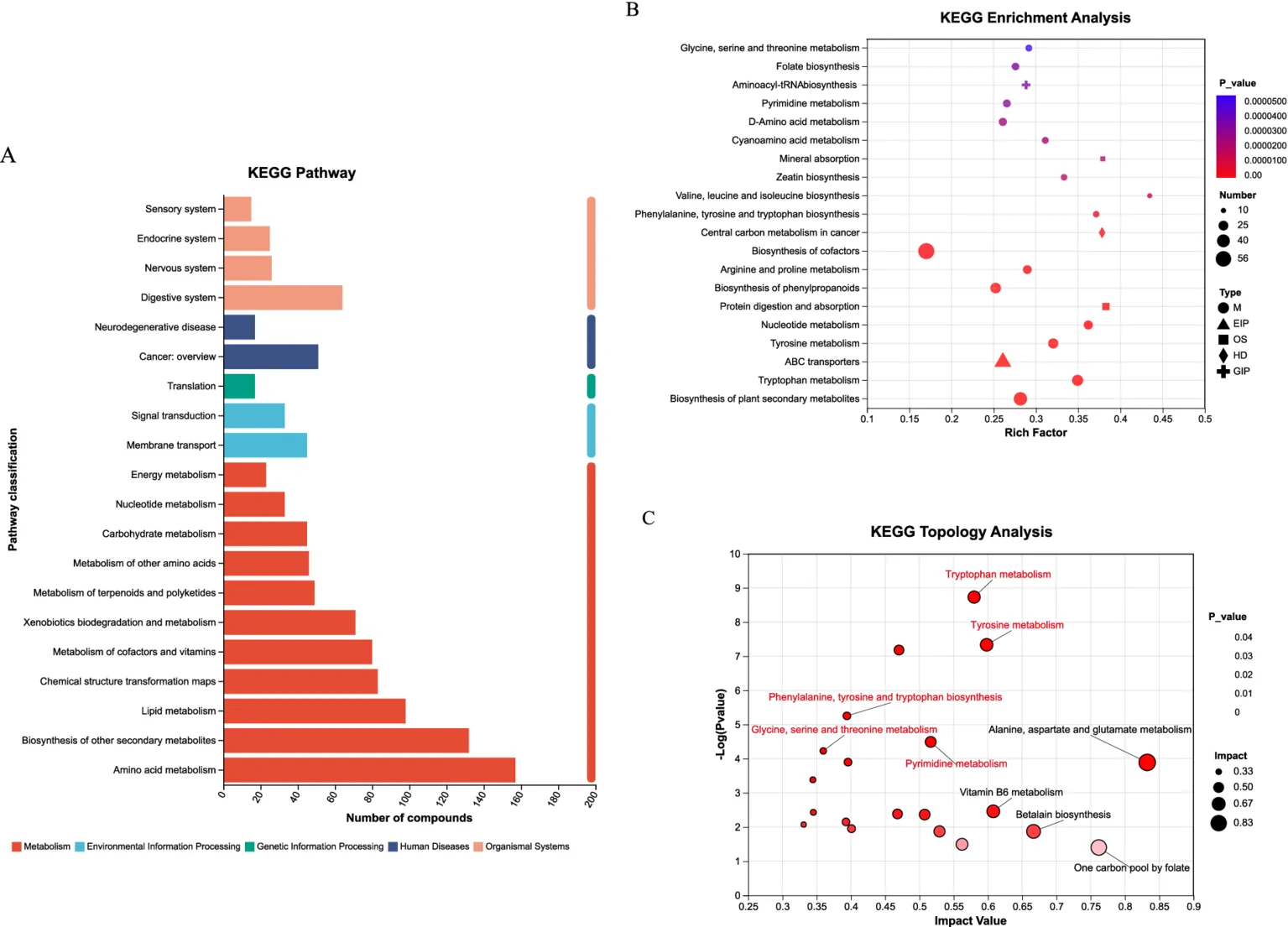
KEGG pathway enrichment and topology analysis of identified metabolites. **(A)** Pathway enrichment analysis based on the KEGG database. **(B)** KEGG enrichment bubble plot. The vertical axis represents KEGG metabolic pathway names, while the horizontal axis shows enrichment factors (Rich factors). Bubble size indicates the number of differentially enriched metabolites within each pathway, and colour denotes *p*-values, with redder colours indicating higher enrichment significance. Different shapes represent distinct KEGG level-1 classifications. **(C)** KEGG pathway topology analysis. The horizontal axis represents pathway impact values calculated based on pathway topology, reflecting the relative importance of metabolites within each pathway, while the vertical axis shows −log10 (*p*-values), with higher values indicating greater statistical significance. Pathways appearing in both the enrichment bubble plot and topology analysis are highlighted in red font, whereas the top 40 pathways with the highest pathway impact values are shown in black font.

KEGG pathway enrichment and topology analyses were performed for all identified metabolites. KEGG pathway enrichment analysis (Figure 6B) revealed significant enrichment of several amino acid metabolism pathways, including arginine and proline metabolism, tyrosine metabolism, tryptophan metabolism, and valine, leucine and isoleucine biosynthesis. In addition, pathways related to ABC transporters, protein digestion and absorption, and mineral absorption were also highly enriched. These findings are partially consistent with the relatively high predicted abundance of several transporter-related KOs identified by PICRUSt2. However, because these results were derived from metabolomics data and 16S rRNA gene-based functional prediction, respectively, their biological relevance requires further validation. Notably, pathways involved in the biosynthesis of plant secondary metabolites were also significantly enriched (Supplementary Table 10).

Metabolic pathway topology analysis was further performed for the identified metabolites (Figure 6C). Alanine, aspartate and glutamate metabolism showed both high enrichment significance and a high pathway impact value. In addition, tryptophan metabolism, tyrosine metabolism, glycine, serine and threonine metabolism, and phenylalanine, tyrosine and tryptophan biosynthesis also exhibited relatively high enrichment significance and pathway impact values in both the enrichment and topology analyses. The remaining enriched pathways are listed in Supplementary Table 11.

### Correlation and network analysis of the fecal microbiota and metabolites in *Rhinolophus ferrumequinum*

To explore the associations between the fecal microbiota and metabolites, Spearman correlation analysis was performed between the top 30 bacterial genera and the top 30 metabolites ranked by relative abundance, followed by hierarchical clustering analysis (Figure 7A). Given the limited sample size, this analysis was intended to identify potential associations between microbial taxa and metabolites. A microbe–metabolite correlation network was constructed using a threshold of |r| > 0.8 and *p* < 0.05 (Figure 7B; Supplementary Table 12). The resulting network consisted of three major correlation modules. *Candidatus Arthromitus* and *Escherichia-Shigella* were significantly positively correlated with taurocholic acid, choline, and N-linoleoyl histidine, whereas *Enterococcus* showed predominantly negative correlations with these metabolites. *Acinetobacter* and *Serratia* were significantly positively correlated with multiple acylcarnitines, forming another correlation module. In addition, *Fusobacterium* was significantly negatively correlated with several long-chain acylcarnitines, such as tetradecanoylcarnitine, whereas *Mycoplasma* showed significant positive correlations with these metabolites.

**Figure 7:**
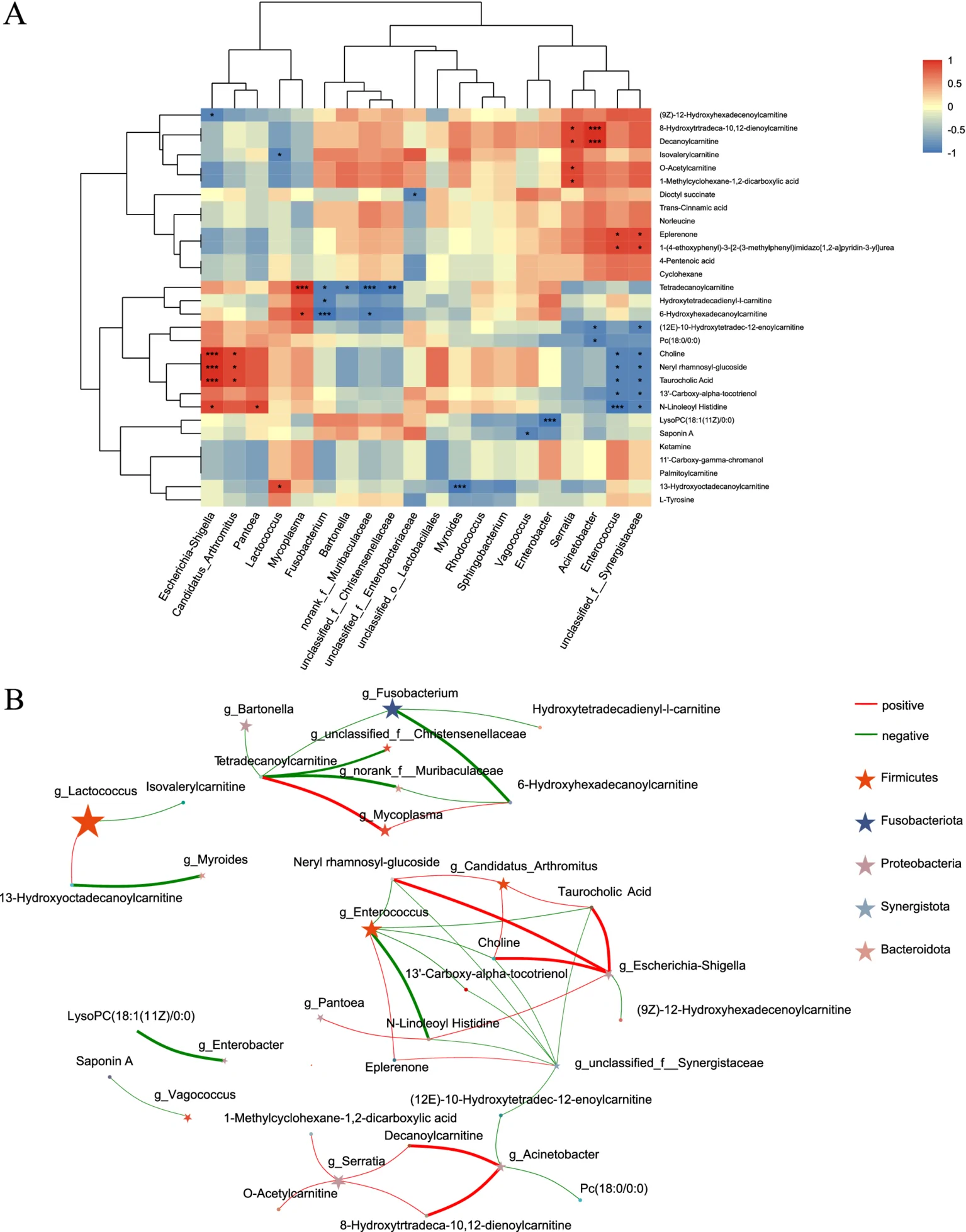
Correlation analysis between fecal microbiota and differential metabolites. **(A)** Heatmap showing Spearman correlations between dominant bacterial genera (columns) and differential metabolites (rows). Red and blue indicate positive and negative correlations, respectively, with color intensity reflecting correlation strength (*r*). Asterisks denote statistical significance (*p < 0.05, ** *p* < 0.01, *** *p* < 0.001). Dendrograms on the top and left represent hierarchical clustering of genera and metabolites. **(B)** Microbe–metabolite co-occurrence network constructed based on significant Spearman correlations (|*r*| > 0.8, *p* < 0.05). Star-shaped nodes represent bacterial genera, colored by phylum (as indicated in the legend), with node size proportional to mean relative abundance. Edges denote significant correlations: red lines indicate positive correlations, whereas green lines indicate negative correlations.

### Integrated analysis of 16S rRNA functional prediction and metabolomics in the tryptophan and tyrosine metabolism pathways

Integrated mapping analysis of the tryptophan metabolism (map00380) and tyrosine metabolism (map00350) pathways was performed by combining 16S rRNA gene-based functional prediction with untargeted metabolomics data (Supplementary Figures 5, 6). In the tryptophan metabolism pathway, L-formylkynurenine, 2-aminomuconate semialdehyde, indole-3-acetaldehyde, indole-3-acetate, 5-hydroxyindoleacetate, and 6-hydroxymelatonin were detected. PICRUSt2 functional prediction also identified functional entries corresponding to enzymes involved in this pathway, including TDO, IDO, KMO, amidase, ASMT, and CYP1A1. In the tyrosine metabolism pathway, homogentisate, L-DOPA, and dopamine were detected and mapped to the corresponding metabolic pathway. PICRUSt2 functional prediction further identified functional entries corresponding to tyrosinase and other related enzymes.

## Discussion

### Community composition and adaptation to the host ecological niche

The gut microbiota of many terrestrial mammals is frequently characterized by the predominance of Bacteroidota and Bacillota, whereas bats often exhibit a relatively higher abundance of Pseudomonadota compared with many other mammalian groups (Carrillo-Araujo et al., 2015). Comparative analyses across vertebrates have shown that bat gut microbiomes share similarities with those of birds, potentially reflecting convergent adaptations associated with powered flight (Song et al., 2020). In the present study, Bacillota and Pseudomonadota were the predominant bacterial phyla, consistent with previous reports on *Rhinolophus ferrumequinum*, although their relative abundances differed among studies (Xiao et al., 2019). The bat gut is characterized by a short intestinal tract, rapid intestinal transit, uneven oxygen distribution, and nocturnal pulse feeding. These characteristics favor the colonization of fast-growing, facultatively anaerobic, and opportunistic bacteria while limiting, to some extent, the establishment of slow-growing obligate anaerobes (Price et al., 2015).

Several bacterial genera detected in the present study have also been reported from *Rhinolophus ferrumequinum* and other insectivorous bats. For instance, lactic acid bacteria, including the genera *Lactococcus* and *Enterococcus*, as well as *Fusobacterium*, were among the dominant bacterial taxa detected in our study and have similarly been reported as highly abundant taxa in other insectivorous bats. In addition, low-abundance genera such as *Serratia, Acinetobacter, and Mycoplasma* have also been detected in the microbial communities of insectivorous bats (Popov et al., 2023; Dai et al., 2024; Unno et al., 2026). Furthermore, a comparative analysis of insectivorous bats identified *Mycoplasma* as a characteristic taxon associated with *Rhinolophus ferrumequinum* (An et al., 2024).

Previous studies have suggested that these lactic acid bacterial taxa may participate in the fermentation of carbohydrates and amino acids, producing metabolites such as lactic acid and short-chain fatty acids, which may contribute to the maintenance of intestinal metabolic homeostasis (Tang et al., 2023; Du et al., 2024). In addition, the relatively high abundance of *Fusobacterium* is consistent with previous reports describing its involvement in protein fermentation and amino acid metabolism in animal gut ecosystems (Teullet et al., 2023; Robinson et al., 2025). Together with the enrichment of lipid- and amino acid-related metabolic pathways observed in the present metabolomic analysis, these microbial characteristics may be associated with nutrient transformation processes linked to the insectivorous dietary niche of *Rhinolophus ferrumequinum*. However, functional interpretations based solely on 16S rRNA gene sequencing should be interpreted with caution and require further validation through metagenomic approaches and experimental studies.

Dietary specialization may represent an important ecological factor shaping bat-associated microbiota. Previous hologenomic analyses of the common vampire bat (*Desmodus rotundus*) showed that specialized diets were associated with distinct microbial functional profiles, suggesting a potential role of microbiota in host dietary adaptation (Zepeda Mendoza et al., 2018). Similarly, comparative analyses of piscivorous and insectivorous bats revealed that dietary niche expansion was accompanied by shifts in gut microbial composition and functional potential, highlighting possible links between microbiota and the utilization of specialized dietary resources (Aizpurua et al., 2021). In addition, *Candidatus Arthromitus* and *Escherichia-Shigella* detected in the present study have also been reported in the non-strictly insectivorous bat *Ia io*, suggesting that certain microbial members may be shaped by multiple factors, including host dietary ecology and environmental exposure (Gong et al., 2021). Collectively, these findings support the hypothesis that dietary ecology may influence the composition and functional potential of bat gut microbiota.

### Functional potential of the gut microbiome and host-microbe interactions

PICRUSt2 functional prediction showed that metabolism-related pathways accounted for a large proportion of the predicted functions in the gut microbiota of *Rhinolophus ferrumequinum*, consistent with previous reports that metabolic functions predominate in the fecal microbiota of most animals. Notably, functional entries associated with the ABC transport system and iron transport exhibited relatively high predicted abundances. Previous studies have shown that the ABC transport system is widely involved in nutrient uptake, metabolite transport, and environmental adaptation in microorganisms, whereas iron transport systems are essential for microbial acquisition of this indispensable micronutrient and play important physiological roles in the host intestinal environment (Tanaka et al., 2018; Murdoch and Skaar, 2022). In addition, pathways related to glycolysis, fermentation, and the biosynthesis of several amino acids were also predicted, suggesting that the gut microbiota of *Rhinolophus ferrumequinum* possesses substantial potential for core metabolic activities (Li et al., 2024). It should be noted that PICRUSt2 infers potential functions from 16S rRNA gene data and does not directly reflect the actual expression of the corresponding genes. Therefore, these predicted functions require further experimental validation.

### Metabolic phenotype adapted to an insectivorous ecological niche

Untargeted metabolomics analysis showed that the fecal metabolic profile of *Rhinolophus ferrumequinum* was primarily composed of lipids, organic acids, and amino acids, with amino acid metabolism pathways showing a high degree of enrichment. *Rhinolophus ferrumequinum* mainly consumes insect prey, particularly Lepidoptera and Coleoptera (Aldasoro et al., 2024). These insect prey generally contain abundant proteins, amino acids, and lipids, which may provide important nutritional inputs for insectivorous bats (Gautam et al., 2025). Therefore, the metabolic characteristics observed in this study may be associated with its insectivorous diet. The gut microbiota may contribute to host lipid utilization by secreting lipases and participating in β-oxidation, thereby facilitating energy extraction from dietary lipids (Arrese and Soulages, 2010).

KEGG pathway enrichment and topology analyses both identified multiple amino acid metabolism pathways, and the ABC transporter pathway also showed a high level of enrichment, which was partially consistent with the PICRUSt2 functional prediction. In addition, the tryptophan and tyrosine metabolism pathways were highly enriched in the KEGG analysis, and metabolomics analysis detected several compounds related to hormones and neurotransmitters. Previous studies have shown that tryptophan and tyrosine metabolism participate in conserved physiological processes across animals, including neurotransmitter biosynthesis and immune regulation (Connil et al., 2002; Wang et al., 2023).

Interestingly, although *Rhinolophus ferrumequinum* is a strictly insectivorous bat, KEGG analysis identified pathways associated with plant secondary metabolites, including isoflavonoid and phenylpropanoid biosynthesis. These findings do not imply direct plant consumption but may reflect trophic transfer, as insect prey such as Lepidoptera and Coleoptera can acquire plant-derived secondary metabolites and transfer them to higher consumers (Erb and Robert, 2016; Divekar et al., 2022). In addition, plant-derived metabolites may undergo further transformation mediated by host-associated microbiota. Recent studies have suggested that bat-associated microbial communities may contain bacterial taxa potentially involved in the transformation of flavonoid-related compounds (Brändel et al., 2026). Supporting this possibility, our previous study also observed enrichment of isoflavonoid-related metabolites and pathways in mice receiving fecal microbiota from *Hipposideros armiger* (Liu et al., 2022). In other mammalian systems, gut microbiota have been shown to convert isoflavonoids and other aromatic compounds into structurally modified metabolites, such as equol and phenylpropionic acid, which may influence immune regulation, hormone-related pathways, and metabolic homeostasis (Mayo et al., 2019; Bernatoniene et al., 2021; Cho et al., 2023; Horio et al., 2023; Hu et al., 2023). However, the origin and physiological significance of these metabolites could not be determined in the present study and require further investigation through dietary composition analysis, metagenomics, and functional experiments.

### Microbial-metabolite associations reveal potential metabolic interactions

Correlation analysis revealed significant associations between dominant bacterial genera and specific metabolites, suggesting potential links between microbial community composition and host-associated metabolic profiles. In the present study, *Serratia*, *Acinetobacter*, and *Mycoplasma* were significantly positively correlated with multiple acylcarnitines. Previous studies have suggested that members of these genera may possess capacities related to lipid utilization and metabolism (Rodwall, 1968; Johnson et al., 2015; Godoy et al., 2022). Therefore, these taxa may potentially contribute to, or respond to, intestinal lipid metabolic processes in *Rhinolophus ferrumequinum*.

In addition, *Escherichia-Shigella* and *Candidatus Arthromitus* were positively correlated with choline and taurocholic acid, whereas *Enterococcus* and *Fusobacterium* showed negative correlations with these metabolites. The observed associations between bacterial taxa and lipid-related metabolites may provide insights into the potential interaction between microbial composition and nutrient metabolism in *Rhinolophus ferrumequinum*. Taurocholic acid, a conjugated bile acid, plays an essential role in dietary lipid emulsification and absorption, and bile acid metabolism is closely linked to gut microbial activity and host lipid homeostasis (Collins et al., 2023; He et al., 2024). Meanwhile, acylcarnitines represent intermediates involved in fatty acid transport and mitochondrial β-oxidation, suggesting that their correlations with bacterial taxa may reflect potential links between microbial composition and lipid metabolic status. Previous studies have shown that these genera differ in their tolerance to bile acids and their capacity for nutrient utilization (Joffre et al., 2019; Oemcke et al., 2021; Zhang et al., 2022; Fujii et al., 2025; Incognito et al., 2025). Therefore, the correlations observed in the present study may reflect distinct adaptive strategies of different microbial taxa to the bat gut microenvironment. However, given the limited sample size and the fact that correlation analysis does not establish causality, further genomic and functional validation is required to clarify whether these bacterial taxa are functionally involved in host lipid-related metabolic processes.

### Potential biological significance of tryptophan and tyrosine metabolism

In the present study, the tryptophan and tyrosine metabolism pathways showed a high degree of enrichment in the metabolomics analysis, and the functional prediction also identified corresponding metabolic pathways, suggesting that these two amino acid metabolism pathways may represent important components of the gut microecology of *Rhinolophus ferrumequinum*. Metabolites associated with the kynurenine pathway were detected, and functional prediction indicated potential microbial functions related to enzymes involved in tryptophan catabolism, including TDO- and IDO-related activities. Previous studies have shown that kynurenine metabolism is closely associated with host immune regulation, redox homeostasis, and intestinal microenvironment maintenance (Zelante et al., 2013; Yoshioka et al., 2022; Chajadine et al., 2024; Wu et al., 2024). In addition, several indole metabolites were detected in this study. These metabolites have been suggested to be involved in microbial–host metabolic interactions, and their reported roles in immune regulation and intestinal homeostasis provide potential biological relevance (Li et al., 2021). The gut microbiota may therefore help bats maintain a low-inflammatory state despite sustained exposure to pathogens. Furthermore, the detection of serotonin and melatonin precursors together with the predicted corresponding enzymes may suggest an indirect role of the gut microbiota in circadian rhythm regulation (Haduch et al., 2023). Recent studies have demonstrated that gut microbiota exhibit diurnal oscillations and may interact with host circadian physiology through microbial metabolites (Schmid et al., 2023; Melville et al., 2025a). Given the nocturnal lifestyle and rhythmic feeding behavior of bats, these microbial metabolic features may represent potential mechanisms linking gut microbiota with host physiological adaptation.

Tyrosine metabolism was another prominent metabolic pathway identified in this study. In addition to the detection of related metabolites, functional prediction indicated the potential presence of reactions involved in tyrosine metabolism, suggesting that tyrosine-related metabolism represents an important feature of the gut metabolic profile. Previous studies have shown that tyrosine metabolism is involved not only in amino acid catabolism but also in the biosynthesis of various neuroactive metabolites (Shin and Bang, 2025). Catecholamine-related metabolites, including dopamine, were detected in the present study. Together with previous reports that gut bacteria can participate in aromatic amino acid metabolism, these findings suggest that microbial activity may be associated with the transformation of tyrosine-derived metabolites (Maini Rekdal et al., 2019; van Kessel et al., 2019; El-Shehawy et al., 2021; Zou et al., 2024). However, because the present study was based on functional prediction and metabolite detection, the specific microbial contributors, metabolic pathways, and physiological roles of these metabolites in *Rhinolophus ferrumequinum* remain to be determined.

Several limitations should be acknowledged. The relatively small sample size (*n* = 5) and limited availability of detailed host metadata may constrain the assessment of factors underlying inter-individual microbiome variation. Although fecal sampling provides a practical and non-invasive approach for studying wild bats, it may not fully capture the spatial heterogeneity of the gastrointestinal microbiota (Sun et al., 2020; Turjeman et al., 2023). Furthermore, while 16S rRNA gene sequencing and untargeted metabolomics revealed microbial and metabolic characteristics associated with *Rhinolophus ferrumequinum*, future studies integrating larger cohorts, metagenomic approaches, targeted metabolomics, and experimental validation will be important for further elucidating host–microbiota interactions.

## Conclusion

This study characterized the fecal microbial community and fecal metabolic profiles of *Rhinolophus ferrumequinum* using 16S rRNA gene sequencing and untargeted metabolomics, and explored the associations between microbial taxa and metabolites. The fecal microbial community of *Rhinolophus ferrumequinum* was characterized by the predominance of *Lactococcus*, *Fusobacterium*, and *Enterococcus*. Integrated microbiome and metabolome analyses revealed that the fecal metabolic profile exhibited signatures associated with lipid- and amino acid-related metabolic processes, while several bacterial genera, including *Acinetobacter*, *Serratia*, *Fusobacterium*, and *Mycoplasma*, showed significant correlations with lipid-associated metabolites, particularly acylcarnitines and bile acid-related metabolites. These findings expand our understanding of gut microbial ecology and associated metabolic characteristics in insectivorous bats and suggest that gut microbiota may be involved in metabolic processes related to insectivorous dietary ecology. This study provides a foundation for further investigating microbiota–metabolite interactions in bats. Future studies integrating larger cohorts, metagenomic approaches, targeted metabolomics, and functional validation will be essential to further elucidate the biological significance of these associations.

## Data Availability

The 16S rRNA gene sequencing data generated in this study have been deposited in the NCBI Sequence Read Archive under BioProject accession PRJNA1489443 (https://www.ncbi.nlm.nih.gov/bioproject/PRJNA1489443). The untargeted metabolomics data have been deposited in MetaboLights under accession MTBLS14955 (https://www.ebi.ac.uk/metabolights/editor/MTBLS14955/overview).
