# Supplementary material for: Functional potential of gut microbiota in Rhinolophus ferrumequinum revealed by 16S rRNA sequencing and metabolomics
Source: Front Microbiol. 2026 Sep 23;17:1938216. doi: 10.3389/fmicb.2026.1938216 (PMC13642279; doi:10.3389/fmicb.2026.1938216)
Supplement: Supplementary file 1 [file Supplementary_file_1.docx]

***Supplementary Material***

Supplementary Figure 1. Rarefaction curves of 16S rRNA gene sequences from the gut microbiota of *Rhinolophus ferrumequinum*

The curve illustrates changes in observed species richness (Sobs index) for each sample (RF1–RF5) as the number of sequencing reads increases. The curves rise rapidly at early stages and subsequently approach a plateau, indicating that the current sequencing depth is sufficient to capture the majority of microbial diversity within the gut community.

Supplementary Figure 2. Alpha diversity analysis of fecal microbial communities of *Rhinolophus ferrumequinum*. Shannon and Simpson indices were calculated based on ASV-level abundance profiles to evaluate microbial diversity among individual samples. Bars represent the diversity indices of each bat sample.

Supplementary Figure 3. Alpha diversity analysis of fecal microbial communities of *Rhinolophus ferrumequinum*. Chao and Ace indices were calculated based on ASV-level abundance profiles to evaluate microbial diversity among individual samples. Bars represent the diversity indices of each bat sample.

Supplementary Figure 4. Principal coordinate analysis (PCoA) based on unweighted UniFrac distances.

Supplementary Figure 5. Multi-omics integrated mapping of the tryptophan metabolism pathway

This pathway map integrates PICRUSt2-based functional predictions derived from 16S rRNA gene sequencing of the gut microbiota of *Rhinolophus ferrumequinum* with untargeted metabolomics data. Green boxes indicate enzymes (KOs) predicted to be present in the functional repertoire of the gut microbiome, whereas red circles represent metabolites detected in fecal metabolomic profiling.
The map highlights metabolically active pathways along key branches, particularly the serotonin and kynurenine pathways.

Supplementary Figure 6. Multi-omics integrated mapping of the tyrosine metabolism pathway This pathway map integrates PICRUSt2-based functional predictions derived from 16S rRNA sequencing with untargeted metabolomics data from the gut of *Rhinolophus ferrumequinum*. Green rectangles indicate enzymes (KOs) predicted to be present in the functional repertoire of the gut microbiome, whereas red circles represent metabolites detected in the fecal metabolome.
This map highlights metabolically active branches, including the energy catabolic pathway and the catecholamine biosynthesis pathway.

| Samples | ASVs | Effective Sequences |
| --- | --- | --- |
| RF3 | 324 | 30524 |
| RF2 | 348 | 30524 |
| RF1 | 592 | 30524 |
| RF5 | 408 | 30524 |
| RF4 | 193 | 30524 |

Supplementary Table 1. Summary of valid sequence counts

| ASV | Genus | PC1_r | PC1_R2 | PC2_r | PC2_R2 | Total_contribution |
| --- | --- | --- | --- | --- | --- | --- |
| ASV2161 | Fusobacterium | 0.379 | 0.143 | -0.925 | 0.856 | 0.9995 |
| ASV642 | Fusobacterium | 0.379 | 0.143 | -0.925 | 0.856 | 0.9995 |
| ASV736 | Enterobacteriaceae | 0.429 | 0.184 | -0.903 | 0.815 | 0.9987 |
| ASV3188 | Candidatus_Arthromitus | 0.575 | 0.331 | -0.811 | 0.658 | 0.9885 |
| ASV706 | Serratia | 0.742 | 0.55 | 0.6 | 0.361 | 0.9109 |
| ASV2209 | Serratia | 0.742 | 0.551 | 0.6 | 0.36 | 0.9102 |
| ASV3186 | Serratia | 0.748 | 0.559 | 0.58 | 0.336 | 0.8956 |
| ASV737 | Enterococcus | 0.658 | 0.433 | 0.639 | 0.409 | 0.8417 |
| ASV748 | Lactococcus | -0.789 | 0.622 | 0.126 | 0.016 | 0.6381 |
| ASV731 | Lactococcus | -0.713 | 0.509 | 0.116 | 0.013 | 0.5223 |
| ASV70 | Escherichia-Shigella | -0.652 | 0.426 | -0.254 | 0.064 | 0.4901 |
| ASV2922 | Bartonella | 0.636 | 0.404 | -0.2 | 0.04 | 0.4442 |
| ASV2908 | Lactobacillales | -0.559 | 0.312 | 0.032 | 0.001 | 0.313 |
| ASV2910 | Vagococcus | -0.559 | 0.312 | 0.032 | 0.001 | 0.313 |
| ASV2909 | Candidatus_Arthromitus | -0.524 | 0.274 | -0.089 | 0.008 | 0.2825 |
| ASV5473 | Mycoplasma | -0.476 | 0.227 | 0.095 | 0.009 | 0.2357 |
| ASV683 | Enterococcus | -0.27 | 0.073 | 0.229 | 0.052 | 0.1253 |
| ASV629 | Fusobacterium | 0.046 | 0.002 | 0.339 | 0.115 | 0.1171 |
| ASV734 | Synergistaceae | -0.011 | 0 | 0.287 | 0.083 | 0.0827 |
| ASV3196 | Christensenellaceae | -0.043 | 0.002 | 0.261 | 0.068 | 0.07 |

Supplementary Table 2. The contribution of individual ASVs was evaluated based on squared Pearson correlation coefficients (R²) between ASV abundance and PCoA axis scores.

| ASV | Genus | PC1_r | PC1_R2 | PC2_r | PC2_R2 | Total_contribution |
| --- | --- | --- | --- | --- | --- | --- |
| ASV3186 | Serratia | 0.185 | 0.034 | 0.963 | 0.926 | 0.9606 |
| ASV2209 | Serratia | 0.193 | 0.037 | 0.956 | 0.914 | 0.9519 |
| ASV706 | Serratia | 0.194 | 0.038 | 0.956 | 0.914 | 0.9516 |
| ASV737 | Enterococcus | 0.698 | 0.487 | 0.634 | 0.402 | 0.8884 |
| ASV748 | Lactococcus | -0.738 | 0.545 | -0.404 | 0.164 | 0.7081 |
| ASV3196 | unclassified_f__Christensenellaceae | 0.747 | 0.558 | -0.316 | 0.100 | 0.6583 |
| ASV734 | unclassified_f__Synergistaceae | 0.760 | 0.578 | -0.278 | 0.077 | 0.6550 |
| ASV629 | Fusobacterium | 0.780 | 0.609 | -0.201 | 0.040 | 0.6489 |
| ASV2922 | Bartonella | 0.724 | 0.524 | -0.112 | 0.013 | 0.5367 |
| ASV683 | Enterococcus | 0.527 | 0.278 | -0.502 | 0.253 | 0.5302 |
| ASV70 | Escherichia-Shigella | -0.692 | 0.479 | -0.196 | 0.038 | 0.5169 |
| ASV5473 | Mycoplasma | -0.650 | 0.423 | -0.265 | 0.070 | 0.4928 |
| ASV2908 | unclassified_o__Lactobacillales | -0.473 | 0.223 | 0.013 | 0.000 | 0.2236 |
| ASV2910 | Vagococcus | -0.473 | 0.223 | 0.013 | 0.000 | 0.2236 |
| ASV2909 | Candidatus_Arthromitus | -0.442 | 0.195 | -0.039 | 0.002 | 0.1966 |
| ASV2161 | Fusobacterium | 0.214 | 0.046 | -0.334 | 0.112 | 0.1575 |
| ASV642 | Fusobacterium | 0.214 | 0.046 | -0.334 | 0.112 | 0.1575 |
| ASV736 | unclassified_f__Enterobacteriaceae | 0.228 | 0.052 | -0.280 | 0.079 | 0.1307 |
| ASV731 | Lactococcus | -0.190 | 0.036 | -0.276 | 0.076 | 0.1125 |
| ASV3188 | Candidatus_Arthromitus | 0.254 | 0.064 | -0.106 | 0.011 | 0.0754 |

Supplementary Table 3. The contribution of individual ASVs was evaluated based on squared Pearson correlation coefficients (R²) between ASV abundance and PCoA axis scores.

| Phylum | Rf1 | Rf2 | Rf3 | Rf4 | Rf5 | Mean Abundance |
| --- | --- | --- | --- | --- | --- | --- |
| Firmicutes | 0.15149 | 0.56968 | 0.81451 | 0.85434 | 0.19896 | 0.51780 |
| Proteobacteria | 0.52582 | 0.16322 | 0.15401 | 0.13822 | 0.32306 | 0.26086 |
| Fusobacteriota | 0.02185 | 0.14005 | 0.00292 | 0.00023 | 0.36479 | 0.10597 |
| Bacteroidota | 0.14412 | 0.06300 | 0.00423 | 0.00170 | 0.05016 | 0.05264 |
| Actinobacteriota | 0.12403 | 0.01366 | 0.01674 | 0.00311 | 0.02683 | 0.03688 |
| Synergistota | 0.00557 | 0.04501 | 0.00000 | 0.00000 | 0.01353 | 0.01282 |
| Patescibacteria | 0.02392 | 0.00085 | 0.00390 | 0.00141 | 0.00334 | 0.00668 |
| Desulfobacterota | 0.00066 | 0.00147 | 0.00013 | 0.00000 | 0.00770 | 0.00199 |
| Verrucomicrobiota | 0.00016 | 0.00026 | 0.00010 | 0.00000 | 0.00652 | 0.00141 |
| Cyanobacteria | 0.00010 | 0.00161 | 0.00092 | 0.00007 | 0.00046 | 0.00063 |
| Campilobacterota | 0.00000 | 0.00000 | 0.00010 | 0.00000 | 0.00252 | 0.00052 |
| Chloroflexi | 0.00046 | 0.00013 | 0.00108 | 0.00003 | 0.00036 | 0.00041 |
| unclassified_k__norank_d__Bacteria | 0.00092 | 0.00102 | 0.00000 | 0.00007 | 0.00000 | 0.00040 |
| Acidobacteriota | 0.00023 | 0.00000 | 0.00056 | 0.00026 | 0.00010 | 0.00023 |
| Rs-K70_termite_group | 0.00023 | 0.00000 | 0.00000 | 0.00000 | 0.00085 | 0.00022 |
| Nitrospirota | 0.00007 | 0.00003 | 0.00033 | 0.00016 | 0.00029 | 0.00018 |
| Planctomycetota | 0.00013 | 0.00000 | 0.00020 | 0.00003 | 0.00000 | 0.00007 |
| Bdellovibrionota | 0.00003 | 0.00000 | 0.00010 | 0.00020 | 0.00000 | 0.00007 |
| NB1-j | 0.00000 | 0.00000 | 0.00013 | 0.00000 | 0.00016 | 0.00006 |
| WPS-2 | 0.00000 | 0.00000 | 0.00000 | 0.00010 | 0.00013 | 0.00005 |
| others | 0.00023 | 0.00000 | 0.00007 | 0.00007 | 0.00023 | 0.00012 |

Supplementary Table 4. Relative abundance of microbial communities at the phylum level based on 16S rRNA sequencing

| Genus | RF1 | RF2 | RF3 | RF4 | RF5 | Mean Abundance |
| --- | --- | --- | --- | --- | --- | --- |
| Lactococcus | 0.00819 | 0.253931 | 0.451677 | 0.579184 | 0.052975 | 0.269191 |
| Fusobacterium | 0.021262 | 0.11663 | 0.002686 | 0.000229 | 0.358538 | 0.099869 |
| Enterococcus | 0.086751 | 0.175927 | 0.029714 | 0.076759 | 0.019591 | 0.077749 |
| Serratia | 0.226543 | 0.010975 | 0.005471 | 0.005176 | 0.010385 | 0.05171 |
| Bartonella | 0.049273 | 0.062115 | 0.002654 | 0.045735 | 0.06028 | 0.044011 |
| Candidatus Arthromitus | 0.014284 | 0.002817 | 0.111027 | 0.003538 | 0.069781 | 0.04029 |
| Mycoplasma | 0.000229 | 9.83E-05 | 0.000328 | 0.193422 | 0.000164 | 0.038848 |
| unclassified_o__Lactobacillales | 0.000885 | 0.000491 | 0.133207 | 0 | 0.006552 | 0.028227 |
| unclassified_f__Enterobacteriaceae | 0.017429 | 0.006519 | 0.002817 | 0.010451 | 0.089831 | 0.02541 |
| Escherichia-Shigella | 0.004128 | 0.002948 | 0.067684 | 0.016577 | 0.02254 | 0.022776 |
| Acinetobacter | 0.03643 | 0.014677 | 0.014382 | 0.00036 | 0.014284 | 0.016027 |
| Vagococcus | 0.001966 | 0 | 0.077775 | 0 | 0 | 0.015948 |
| unclassified_f__Christensenellaceae | 0.004292 | 0.063163 | 0 | 0 | 0.009894 | 0.01547 |
| Pantoea | 0.004488 | 0.003243 | 0.00498 | 0.003276 | 0.053728 | 0.013943 |
| Sphingobacterium | 0.065391 | 0 | 0.000754 | 0 | 0.000262 | 0.013281 |
| norank_f__Muribaculaceae | 0.001441 | 0.035382 | 3.28E-05 | 0 | 0.028568 | 0.013085 |
| unclassified_f__Synergistaceae | 0.005569 | 0.045014 | 0 | 0 | 0 | 0.010117 |
| Myroides | 0.045374 | 0.000295 | 0.000328 | 0 | 0.000459 | 0.009291 |
| Enterobacter | 0.012187 | 0.005373 | 0.01032 | 0.006945 | 0.002129 | 0.007391 |
| Rhodococcus | 0.03014 | 0.00059 | 0.002293 | 0.000819 | 0.001376 | 0.007044 |
| others | 0.363747 | 0.19981 | 0.08187 | 0.057529 | 0.198663 | 0.180324 |

Supplementary Table 5. Relative abundance of microbial genera based on 16S rRNA sequencing

| Level2 | Level1 | RF1 | RF2 | RF3 | RF4 | RF5 | Mean |
| --- | --- | --- | --- | --- | --- | --- | --- |
| Global and overview maps | Metabolism | 36990582 | 26444425 | 23631662 | 25743073 | 30725057 | 143534798.6 |
| Carbohydrate metabolism | Metabolism | 8894670 | 7093176 | 6896510 | 6813658 | 8090747 | 37788760.31 |
| Amino acid metabolism | Metabolism | 7240751 | 4397435 | 3633134 | 4289609 | 5106201 | 24667130.88 |
| Membrane transport | Environmental Information Processing | 4101620 | 3364479 | 3222571 | 3162171 | 3951905 | 17802746.73 |
| Energy metabolism | Metabolism | 3807698 | 2602975 | 2241318 | 2345102 | 3208928 | 14206020.95 |
| Metabolism of cofactors and vitamins | Metabolism | 3786389 | 2591108 | 1998693 | 2264844 | 3399128 | 14040162.32 |
| Translation | Genetic Information Processing | 2181812 | 2283408 | 2149767 | 2198027 | 2335149 | 11148163.02 |
| Nucleotide metabolism | Metabolism | 2293276 | 1961048 | 1860715 | 1895806 | 2070709 | 10081554.11 |
| Replication and repair | Genetic Information Processing | 2121460 | 1989612 | 1873559 | 1896728 | 2184577 | 10065935.35 |
| Signal transduction | Environmental Information Processing | 2716960 | 1548039 | 1512936 | 1492785 | 1995269 | 9265988.75 |
| Cellular community - prokaryotes | Cellular Processes | 2456314 | 1617208 | 1311444 | 1483608 | 2193957 | 9062530.61 |
| Lipid metabolism | Metabolism | 2120231 | 1389585 | 1365715 | 1408698 | 1528936 | 7813165.31 |
| Xenobiotics biodegradation and metabolism | Metabolism | 1831456 | 917523.7 | 901528.8 | 944397.2 | 1183087 | 5777992.03 |
| Metabolism of other amino acids | Metabolism | 1613732 | 981436.3 | 892703 | 948314.7 | 1272275 | 5708461.67 |
| Glycan biosynthesis and metabolism | Metabolism | 1250403 | 989089.6 | 921644.8 | 941564.1 | 1153011 | 5255712.84 |
| Biosynthesis of other secondary metabolites | Metabolism | 1313460 | 917651.7 | 757720.9 | 837526.5 | 1126536 | 4952895.37 |
| Folding, sorting and degradation | Genetic Information Processing | 1133679 | 933443.9 | 842998.8 | 881332.8 | 1054002 | 4845456.83 |
| Drug resistance: antimicrobial | Human Diseases | 1039802 | 719234.5 | 756609.1 | 780585.2 | 844134 | 4140364.88 |
| Metabolism of terpenoids and polyketides | Metabolism | 940402.9 | 672340.3 | 624266.7 | 633682.2 | 766190.5 | 3636882.62 |
| Infectious disease: bacterial | Human Diseases | 713381.8 | 527636.8 | 518650.5 | 509519.1 | 625694.7 | 2894882.9 |
| Cell growth and death | Cellular Processes | 649252.9 | 529467.4 | 428097.8 | 485340.7 | 565363.3 | 2657522.08 |
| Cell motility | Cellular Processes | 849860.5 | 436682.9 | 221940 | 365149.5 | 771761.7 | 2645394.68 |
| Endocrine system | Organismal Systems | 571335.2 | 414836.4 | 418189.4 | 435650.4 | 435805.8 | 2275817.21 |
| Cancer: overview | Human Diseases | 557602 | 376860.4 | 337212.2 | 364015.1 | 428246.1 | 2063935.77 |
| Aging | Organismal Systems | 451640.5 | 238997.1 | 227451.3 | 234500.2 | 279953.4 | 1432542.54 |
| Drug resistance: antineoplastic | Human Diseases | 275022 | 189305.6 | 160476.2 | 181280.1 | 219659 | 1025742.92 |
| Neurodegenerative disease | Human Diseases | 342116.5 | 167801.6 | 150641.2 | 159879.8 | 202113.7 | 1022552.83 |
| Transport and catabolism | Cellular Processes | 286404.5 | 131784.3 | 123221.4 | 133702.3 | 130969.8 | 806082.31 |
| Environmental adaptation | Organismal Systems | 239444.2 | 135055.2 | 99423.9 | 111145.2 | 180362.1 | 765430.64 |
| Endocrine and metabolic disease | Human Diseases | 182463.7 | 144763.2 | 127101.3 | 141914.9 | 155625.9 | 751868.89 |
| Cardiovascular disease | Human Diseases | 203266.4 | 112420.9 | 69528.14 | 88487.68 | 150865.3 | 624568.48 |
| Transcription | Genetic Information Processing | 107577.5 | 126581.7 | 125772.7 | 127317.3 | 128417.6 | 615666.8 |
| Nervous system | Organismal Systems | 141112.5 | 111370.3 | 83825.19 | 89896.32 | 123247.6 | 549451.94 |
| Immune system | Organismal Systems | 146517.1 | 96026.38 | 49428.24 | 62289.27 | 157008.6 | 511269.51 |
| Infectious disease: viral | Human Diseases | 176165.2 | 96820.57 | 49136.5 | 71398.31 | 101342 | 494862.51 |
| Cancer: specific types | Human Diseases | 185110.2 | 68601.48 | 32913.83 | 45688.75 | 110926.2 | 443240.44 |
| Infectious disease: parasitic | Human Diseases | 85390.45 | 36035.29 | 34302.66 | 39262.68 | 44348.44 | 239339.52 |
| Immune disease | Human Diseases | 40902.41 | 37743.66 | 42731 | 46842.54 | 37076.45 | 205296.06 |
| Digestive system | Organismal Systems | 53206.81 | 31330.99 | 37790.07 | 45742.89 | 31703.87 | 199774.63 |
| Excretory system | Organismal Systems | 38893.25 | 14492.01 | 6122.24 | 6035.85 | 28767.15 | 94310.5 |
| Development and regeneration | Organismal Systems | 16568.29 | 13857.69 | 4101.55 | 6767.75 | 24079.66 | 65374.94 |
| Circulatory system | Organismal Systems | 22581.63 | 8517 | 3701.3 | 5328 | 7830 | 47957.93 |
| Substance dependence | Human Diseases | 9881.01 | 1438.5 | 2083.74 | 705 | 1983.66 | 16091.91 |
| Cellular community - eukaryotes | Cellular Processes | 18.7 | 14 | 92.2 | 5 | 34 | 163.9 |
| Signaling molecules and interaction | Environmental Information Processing | 31 | 10 | 58 | 6 | 42.99 | 147.99 |

Supplementary Table 6. KEGG pathway abundance of predicted gut microbial functions based on 16S rRNA sequencing

| Superclass | Number |
| --- | --- |
| Lipids and lipid-like molecules | 597 |
| Organic acids and derivatives | 587 |
| Organoheterocyclic compounds | 398 |
| Organic oxygen compounds | 220 |
| Benzenoids | 195 |
| Phenylpropanoids and polyketides | 127 |
| Nucleosides, nucleotides, and analogues | 63 |
| Not Available | 42 |
| Organic nitrogen compounds | 38 |
| Alkaloids and derivatives | 27 |
| Lignans, neolignans and related compounds | 6 |
| Hydrocarbons | 3 |
| Organosulfur compounds | 3 |
| Homogeneous non-metal compounds | 2 |
| Hydrocarbon derivatives | 1 |

Supplementary Table 7. Classification of compounds based on the HMDB database

| First Category | Second Category | Number |
| --- | --- | --- |
| Antibiotics | Aminoglycosides | 4 |
| Antibiotics | Others | 1 |
| Antibiotics | beta-Lactams | 3 |
| Carbohydrates | Monosaccharides | 13 |
| Carbohydrates | Oligosaccharides | 2 |
| Hormones and transmitters | Neurotransmitters | 9 |
| Hormones and transmitters | Other hormones | 4 |
| Hormones and transmitters | Peptide hormones | 2 |
| Hormones and transmitters | Steroid hormones | 5 |
| Lipids | Eicosanoids | 7 |
| Lipids | Fatty acids | 10 |
| Lipids | Phospholipids | 13 |
| Nucleic acids | Bases | 5 |
| Nucleic acids | Nucleosides | 6 |
| Nucleic acids | Nucleotides | 6 |
| Organic acids | Carboxylic acids | 17 |
| Peptides | Amines | 3 |
| Peptides | Amino acids | 22 |
| Peptides | Peptides | 1 |
| Steroids | 19-Carbon atoms | 3 |
| Steroids | 21-Carbon atoms | 2 |
| Steroids | 23-Carbon atoms | 1 |
| Steroids | 24-Carbon atoms | 7 |
| Vitamins and cofactors | Cofactors | 5 |
| Vitamins and cofactors | Vitamins | 6 |

Supplementary Table 8. Classification of compounds based on the KEGG database

| First Category | Second Category | Number |
| --- | --- | --- |
| Cellular Processes | Cell growth and death | 5 |
| Cellular Processes | Cell motility | 3 |
| Cellular Processes | Cellular community - eukaryotes | 8 |
| Cellular Processes | Cellular community - prokaryotes | 5 |
| Drug Development | Chronology: Antiinfectives | 3 |
| Drug Development | Skeleton-based classification | 1 |
| Drug Development | Target-based classification: Enzymes | 1 |
| Drug Development | Target-based classification: G protein-coupled receptors | 1 |
| Drug Development | Target-based classification: Nuclear receptors | 2 |
| Environmental Information Processing | Membrane transport | 45 |
| Environmental Information Processing | Signal transduction | 33 |
| Environmental Information Processing | Signaling molecules and interaction | 15 |
| Genetic Information Processing | Folding, sorting and degradation | 1 |
| Genetic Information Processing | Translation | 17 |
| Human Diseases | Cancer: overview | 51 |
| Human Diseases | Cancer: specific types | 9 |
| Human Diseases | Cardiovascular disease | 7 |
| Human Diseases | Drug resistance: antimicrobial | 2 |
| Human Diseases | Drug resistance: antineoplastic | 12 |
| Human Diseases | Endocrine and metabolic disease | 12 |
| Human Diseases | Immune disease | 2 |
| Human Diseases | Infectious disease: bacterial | 9 |
| Human Diseases | Infectious disease: parasitic | 8 |
| Human Diseases | Infectious disease: viral | 5 |
| Human Diseases | Neurodegenerative disease | 17 |
| Human Diseases | Substance dependence | 11 |
| Metabolism | Amino acid metabolism | 157 |
| Metabolism | Biosynthesis of other secondary metabolites | 132 |
| Metabolism | Carbohydrate metabolism | 45 |
| Metabolism | Chemical structure transformation maps | 83 |
| Metabolism | Energy metabolism | 23 |
| Metabolism | Glycan biosynthesis and metabolism | 5 |
| Metabolism | Lipid metabolism | 98 |
| Metabolism | Metabolism of cofactors and vitamins | 80 |
| Metabolism | Metabolism of other amino acids | 46 |
| Metabolism | Metabolism of terpenoids and polyketides | 49 |
| Metabolism | Nucleotide metabolism | 33 |
| Metabolism | Xenobiotics biodegradation and metabolism | 71 |
| Organismal Systems | Aging | 2 |
| Organismal Systems | Circulatory system | 9 |
| Organismal Systems | Development and regeneration | 7 |
| Organismal Systems | Digestive system | 64 |
| Organismal Systems | Endocrine system | 25 |
| Organismal Systems | Environmental adaptation | 7 |
| Organismal Systems | Excretory system | 9 |
| Organismal Systems | Immune system | 8 |
| Organismal Systems | Nervous system | 26 |
| Organismal Systems | Sensory system | 15 |

Supplementary Table 9. Classification of compounds based on the KEGG database

| First Category | Second Category | Pathway Description | KEGGID | Enrich Factor | Ratio_in_study | Ratio_in_pop | P |
| --- | --- | --- | --- | --- | --- | --- | --- |
| Metabolism | Carbohydrate metabolism | Glycolysis / Gluconeogenesis | map00010 | 0.03125 | 1/586 | 32/6496 | 0.9518 |
| Metabolism | Lipid metabolism | Fatty acid elongation | map00062 | 0.025 | 1/586 | 40/6496 | 0.9775 |
| Metabolism | Lipid metabolism | Steroid biosynthesis | map00100 | 0.01754386 | 1/586 | 57/6496 | 0.9955 |
| Metabolism | Energy metabolism | Photosynthesis | map00195 | 0.090909091 | 1/586 | 11/6496 | 0.6468 |
| Metabolism | Biosynthesis of other secondary metabolites | Clavulanic acid biosynthesis | map00331 | 0.1 | 1/586 | 10/6496 | 0.6117 |
| Metabolism | Biosynthesis of other secondary metabolites | Prodigiosin biosynthesis | map00333 | 0.055555556 | 1/586 | 18/6496 | 0.8181 |
| Metabolism | Biosynthesis of other secondary metabolites | Benzoxazinoid biosynthesis | map00402 | 0.111111111 | 1/586 | 9/6496 | 0.5732 |
| Metabolism | Biosynthesis of other secondary metabolites | Indole diterpene alkaloid biosynthesis | map00403 | 0.03030303 | 1/586 | 33/6496 | 0.9562 |
| Metabolism | Metabolism of other amino acids | Phosphonate and phosphinate metabolism | map00440 | 0.017857143 | 1/586 | 56/6496 | 0.9951 |
| Metabolism | Metabolism of other amino acids | Selenocompound metabolism | map00450 | 0.037037037 | 1/586 | 27/6496 | 0.9225 |
| Metabolism | Glycan biosynthesis and metabolism | N-Glycan biosynthesis | map00510 | 0.071428571 | 1/586 | 14/6496 | 0.7342 |
| Metabolism | Metabolism of terpenoids and polyketides | Biosynthesis of 12-, 14- and 16-membered macrolides | map00522 | 0.011494253 | 1/586 | 87/6496 | 0.9997 |
| Metabolism | Metabolism of terpenoids and polyketides | Polyketide sugar unit biosynthesis | map00523 | 0.017241379 | 1/586 | 58/6496 | 0.9959 |
| Metabolism | Glycan biosynthesis and metabolism | Lipopolysaccharide biosynthesis | map00540 | 0.018518519 | 1/586 | 54/6496 | 0.9941 |
| Metabolism | Glycan biosynthesis and metabolism | O-Antigen nucleotide sugar biosynthesis | map00541 | 0.010989011 | 1/586 | 91/6496 | 0.9998 |
| Metabolism | Xenobiotics biodegradation and metabolism | Chloroalkane and chloroalkene degradation | map00625 | 0.023255814 | 1/586 | 43/6496 | 0.9831 |
| Metabolism | Xenobiotics biodegradation and metabolism | Ethylbenzene degradation | map00642 | 0.071428571 | 1/586 | 14/6496 | 0.7342 |
| Metabolism | Metabolism of cofactors and vitamins | Thiamine metabolism | map00730 | 0.032258065 | 1/586 | 31/6496 | 0.947 |
| Metabolism | Metabolism of terpenoids and polyketides | Terpenoid backbone biosynthesis | map00900 | 0.02173913 | 1/586 | 46/6496 | 0.9873 |
| Metabolism | Metabolism of terpenoids and polyketides | Insect hormone biosynthesis | map00981 | 0.04 | 1/586 | 25/6496 | 0.9063 |
| Metabolism | Xenobiotics biodegradation and metabolism | Steroid degradation | map00984 | 0.055555556 | 1/586 | 18/6496 | 0.8181 |
| Metabolism | Metabolism of terpenoids and polyketides | Biosynthesis of ansamycins | map01051 | 0.03125 | 1/586 | 32/6496 | 0.9518 |
| Metabolism | Metabolism of terpenoids and polyketides | Nonribosomal peptide structures | map01054 | 0.071428571 | 1/586 | 14/6496 | 0.7342 |
| Metabolism | Metabolism of terpenoids and polyketides | Biosynthesis of type II polyketide products | map01057 | 0.007246377 | 1/586 | 138/6496 | 1 |
| Metabolism | Metabolism of terpenoids and polyketides | Biosynthesis of enediyne antibiotics | map01059 | 0.015873016 | 1/586 | 63/6496 | 0.9975 |
| Human Diseases | Drug resistance: antineoplastic | Endocrine resistance | map01522 | 0.2 | 1/586 | 5/6496 | 0.3768 |
| Environmental Information Processing | Signal transduction | Phosphatidylinositol signaling system | map04070 | 0.034482759 | 1/586 | 29/6496 | 0.9359 |
| Cellular Processes | Cell growth and death | Cell cycle - yeast | map04111 | 0.5 | 1/586 | 2/6496 | 0.1723 |
| Genetic Information Processing | Folding, sorting and degradation | Sulfur relay system | map04122 | 0.090909091 | 1/586 | 11/6496 | 0.6468 |
| Organismal Systems | Endocrine system | Renin-angiotensin system | map04614 | 0.090909091 | 1/586 | 11/6496 | 0.6468 |
| Organismal Systems | Immune system | NOD-like receptor signaling pathway | map04621 | 0.166666667 | 1/586 | 6/6496 | 0.433 |
| Cellular Processes | Cell motility | Regulation of actin cytoskeleton | map04810 | 0.2 | 1/586 | 5/6496 | 0.3768 |
| Organismal Systems | Endocrine system | Glucagon signaling pathway | map04922 | 0.038461538 | 1/586 | 26/6496 | 0.9148 |
| Organismal Systems | Excretory system | Aldosterone-regulated sodium reabsorption | map04960 | 0.125 | 1/586 | 8/6496 | 0.5308 |
| Human Diseases | Neurodegenerative disease | Amyotrophic lateral sclerosis | map05014 | 0.071428571 | 1/586 | 14/6496 | 0.7342 |
| Human Diseases | Neurodegenerative disease | Huntington disease | map05016 | 0.166666667 | 1/586 | 6/6496 | 0.433 |
| Human Diseases | Infectious disease: bacterial | Epithelial cell signaling in Helicobacter pylori infection | map05120 | 0.2 | 1/586 | 5/6496 | 0.3768 |
| Human Diseases | Infectious disease: bacterial | Pertussis | map05133 | 0.1 | 1/586 | 10/6496 | 0.6117 |
| Human Diseases | Infectious disease: bacterial | Tuberculosis | map05152 | 0.1 | 1/586 | 10/6496 | 0.6117 |
| Human Diseases | Cancer: specific types | Renal cell carcinoma | map05211 | 0.333333333 | 1/586 | 3/6496 | 0.247 |
| Human Diseases | Immune disease | Asthma | map05310 | 0.2 | 1/586 | 5/6496 | 0.3768 |
| Drug Development | Chronology: Antiinfectives | Cephalosporins - oral agents | map07013 | 1 | 1/586 | 1/6496 | 0.09021 |
| Drug Development | Skeleton-based classification | Benzoic acid family | map07110 | 0.5 | 1/586 | 2/6496 | 0.1723 |
| Drug Development | Target-based classification: Enzymes | Catecholamine transferase inhibitors | map07216 | 0.2 | 1/586 | 5/6496 | 0.3768 |
| Drug Development | Target-based classification: Nuclear receptors | Glucocorticoid and mineralocorticoid receptor agonists/antagonists | map07225 | 0.25 | 1/586 | 4/6496 | 0.3149 |
| Drug Development | Target-based classification: Nuclear receptors | Progesterone, androgen and estrogen receptor agonists/antagonists | map07226 | 0.2 | 1/586 | 5/6496 | 0.3768 |
| Drug Development | Target-based classification: G protein-coupled receptors | Histamine H2/H3 receptor agonists/antagonists | map07227 | 0.1 | 1/586 | 10/6496 | 0.6117 |
| Metabolism | Carbohydrate metabolism | Citrate cycle (TCA cycle) | map00020 | 0.1 | 2/586 | 20/6496 | 0.5501 |
| Metabolism | Carbohydrate metabolism | Pentose phosphate pathway | map00030 | 0.054054054 | 2/586 | 37/6496 | 0.8595 |
| Metabolism | Xenobiotics biodegradation and metabolism | Chlorocyclohexane and chlorobenzene degradation | map00361 | 0.024096386 | 2/586 | 83/6496 | 0.9965 |
| Metabolism | Xenobiotics biodegradation and metabolism | Bisphenol degradation | map00363 | 0.08 | 2/586 | 25/6496 | 0.6732 |
| Metabolism | Xenobiotics biodegradation and metabolism | Furfural degradation | map00365 | 0.1 | 1/586 | 10/6496 | 0.6117 |
| Metabolism | Carbohydrate metabolism | Starch and sucrose metabolism | map00500 | 0.054054054 | 2/586 | 37/6496 | 0.8595 |
| Metabolism | Biosynthesis of other secondary metabolites | Streptomycin biosynthesis | map00521 | 0.083333333 | 2/586 | 24/6496 | 0.651 |
| Metabolism | Glycan biosynthesis and metabolism | Teichoic acid biosynthesis | map00552 | 0.058823529 | 2/586 | 34/6496 | 0.8251 |
| Metabolism | Carbohydrate metabolism | Inositol phosphate metabolism | map00562 | 0.042553191 | 2/586 | 47/6496 | 0.9341 |
| Metabolism | Lipid metabolism | Ether lipid metabolism | map00565 | 0.08 | 2/586 | 25/6496 | 0.6732 |
| Metabolism | Carbohydrate metabolism | Pyruvate metabolism | map00620 | 0.0625 | 2/586 | 32/6496 | 0.7982 |
| Metabolism | Xenobiotics biodegradation and metabolism | Xylene degradation | map00622 | 0.052631579 | 2/586 | 38/6496 | 0.8695 |
| Metabolism | Xenobiotics biodegradation and metabolism | Atrazine degradation | map00791 | 0.086956522 | 2/586 | 23/6496 | 0.6276 |
| Metabolism | Metabolism of cofactors and vitamins | Retinol metabolism | map00830 | 0.08 | 2/586 | 25/6496 | 0.6732 |
| Metabolism | Metabolism of terpenoids and polyketides | Pinene, camphor and geraniol degradation | map00907 | 0.054545455 | 3/586 | 55/6496 | 0.8849 |
| Metabolism | Metabolism of terpenoids and polyketides | Sesquiterpenoid and triterpenoid biosynthesis | map00909 | 0.02247191 | 2/586 | 89/6496 | 0.9979 |
| Metabolism | Energy metabolism | Nitrogen metabolism | map00910 | 0.105263158 | 2/586 | 19/6496 | 0.5218 |
| Metabolism | Biosynthesis of other secondary metabolites | Stilbenoid, diarylheptanoid and gingerol biosynthesis | map00945 | 0.08 | 2/586 | 25/6496 | 0.6732 |
| Metabolism | Xenobiotics biodegradation and metabolism | Drug metabolism - other enzymes | map00983 | 0.038461538 | 2/586 | 52/6496 | 0.9555 |
| Metabolism | Metabolism of terpenoids and polyketides | Biosynthesis of vancomycin group antibiotics | map01055 | 0.105263158 | 2/586 | 19/6496 | 0.5218 |
| Human Diseases | Drug resistance: antimicrobial | Vancomycin resistance | map01502 | 0.083333333 | 2/586 | 24/6496 | 0.651 |
| Cellular Processes | Cell motility | Bacterial chemotaxis | map02030 | 0.166666667 | 1/586 | 6/6496 | 0.433 |
| Organismal Systems | Endocrine system | PPAR signaling pathway | map03320 | 0.4 | 2/586 | 5/6496 | 0.0676 |
| Environmental Information Processing | Signal transduction | mTOR signaling pathway | map04150 | 0.25 | 1/586 | 4/6496 | 0.3149 |
| Environmental Information Processing | Signal transduction | PI3K-Akt signaling pathway | map04151 | 0.25 | 1/586 | 4/6496 | 0.3149 |
| Environmental Information Processing | Signal transduction | AMPK signaling pathway | map04152 | 0.045454545 | 1/586 | 22/6496 | 0.8755 |
| Organismal Systems | Aging | Longevity regulating pathway | map04211 | 0.125 | 1/586 | 8/6496 | 0.5308 |
| Organismal Systems | Nervous system | GABAergic synapse | map04727 | 0.222222222 | 2/586 | 9/6496 | 0.1918 |
| Organismal Systems | Sensory system | Olfactory transduction | map04740 | 0.125 | 1/586 | 8/6496 | 0.5308 |
| Organismal Systems | Digestive system | Cholesterol metabolism | map04979 | 0.2 | 2/586 | 10/6496 | 0.2262 |
| Human Diseases | Substance dependence | Nicotine addiction | map05033 | 0.285714286 | 2/586 | 7/6496 | 0.1259 |
| Human Diseases | Infectious disease: parasitic | Leishmaniasis | map05140 | 0.166666667 | 1/586 | 6/6496 | 0.433 |
| Human Diseases | Cancer: overview | Chemical carcinogenesis - receptor activation | map05207 | 0.068965517 | 2/586 | 29/6496 | 0.7509 |
| Human Diseases | Cancer: specific types | Prostate cancer | map05215 | 0.181818182 | 2/586 | 11/6496 | 0.261 |
| Human Diseases | Immune disease | Systemic lupus erythematosus | map05322 | 0.333333333 | 1/586 | 3/6496 | 0.247 |
| Drug Development | Chronology: Antiinfectives | Penicillins | map07011 | 1 | 2/586 | 2/6496 | 0.008125 |
| Drug Development | Chronology: Antiinfectives | Cephalosporins - parenteral agents | map07012 | 0.5 | 2/586 | 4/6496 | 0.0431 |
| Metabolism | Energy metabolism | Oxidative phosphorylation | map00190 | 0.1875 | 3/586 | 16/6496 | 0.17 |
| Metabolism | Biosynthesis of other secondary metabolites | Penicillin and cephalosporin biosynthesis | map00311 | 0.166666667 | 3/586 | 18/6496 | 0.2177 |
| Metabolism | Biosynthesis of other secondary metabolites | Staurosporine biosynthesis | map00404 | 0.0625 | 3/586 | 48/6496 | 0.8208 |
| Metabolism | Lipid metabolism | Glycerolipid metabolism | map00561 | 0.078947368 | 3/586 | 38/6496 | 0.6793 |
| Metabolism | Xenobiotics biodegradation and metabolism | Dioxin degradation | map00621 | 0.045454545 | 3/586 | 66/6496 | 0.945 |
| Metabolism | Carbohydrate metabolism | Propanoate metabolism | map00640 | 0.073170732 | 3/586 | 41/6496 | 0.7288 |
| Metabolism | Metabolism of cofactors and vitamins | One carbon pool by folate | map00670 | 0.333333333 | 3/586 | 9/6496 | 0.04061 |
| Metabolism | Biosynthesis of other secondary metabolites | Indole alkaloid biosynthesis | map00901 | 0.037037037 | 3/586 | 81/6496 | 0.9812 |
| Metabolism | Energy metabolism | Sulfur metabolism | map00920 | 0.090909091 | 3/586 | 33/6496 | 0.5826 |
| Metabolism | Biosynthesis of other secondary metabolites | Anthocyanin biosynthesis | map00942 | 0.045454545 | 3/586 | 66/6496 | 0.945 |
| Metabolism | Metabolism of terpenoids and polyketides | Type I polyketide structures | map01052 | 0.12 | 3/586 | 25/6496 | 0.3953 |
| Metabolism | Metabolism of terpenoids and polyketides | Biosynthesis of siderophore group nonribosomal peptides | map01053 | 0.125 | 3/586 | 24/6496 | 0.3699 |
| Environmental Information Processing | Signal transduction | FoxO signaling pathway | map04068 | 0.4 | 2/586 | 5/6496 | 0.0676 |
| Organismal Systems | Endocrine system | Ovarian steroidogenesis | map04913 | 0.125 | 3/586 | 24/6496 | 0.3699 |
| Organismal Systems | Endocrine system | Prolactin signaling pathway | map04917 | 0.272727273 | 3/586 | 11/6496 | 0.0697 |
| Organismal Systems | Excretory system | Proximal tubule bicarbonate reclamation | map04964 | 0.176470588 | 3/586 | 17/6496 | 0.1935 |
| Metabolism | Lipid metabolism | Fatty acid degradation | map00071 | 0.08 | 4/586 | 50/6496 | 0.6725 |
| Metabolism | Lipid metabolism | Secondary bile acid biosynthesis | map00121 | 0.111111111 | 4/586 | 36/6496 | 0.4107 |
| Metabolism | Biosynthesis of other secondary metabolites | Caffeine metabolism | map00232 | 0.181818182 | 4/586 | 22/6496 | 0.1308 |
| Metabolism | Biosynthesis of other secondary metabolites | Carbapenem biosynthesis | map00332 | 0.125 | 4/586 | 32/6496 | 0.3259 |
| Metabolism | Biosynthesis of other secondary metabolites | Novobiocin biosynthesis | map00401 | 0.125 | 4/586 | 32/6496 | 0.3259 |
| Metabolism | Lipid metabolism | Sphingolipid metabolism | map00600 | 0.114285714 | 4/586 | 35/6496 | 0.3896 |
| Metabolism | Xenobiotics biodegradation and metabolism | Polycyclic aromatic hydrocarbon degradation | map00624 | 0.037383178 | 4/586 | 107/6496 | 0.9899 |
| Metabolism | Xenobiotics biodegradation and metabolism | Naphthalene degradation | map00626 | 0.066666667 | 4/586 | 60/6496 | 0.8028 |
| Metabolism | Carbohydrate metabolism | C5-Branched dibasic acid metabolism | map00660 | 0.142857143 | 5/586 | 35/6496 | 0.204 |
| Metabolism | Energy metabolism | Carbon fixation pathways in prokaryotes | map00720 | 0.090909091 | 4/586 | 44/6496 | 0.5702 |
| Metabolism | Metabolism of terpenoids and polyketides | Limonene degradation | map00903 | 0.085106383 | 4/586 | 47/6496 | 0.6235 |
| Metabolism | Metabolism of terpenoids and polyketides | Carotenoid biosynthesis | map00906 | 0.032520325 | 4/586 | 123/6496 | 0.9968 |
| Environmental Information Processing | Signal transduction | cGMP-PKG signaling pathway | map04022 | 0.2 | 2/586 | 10/6496 | 0.2262 |
| Cellular Processes | Cell growth and death | Ferroptosis | map04216 | 0.137931034 | 4/586 | 29/6496 | 0.263 |
| Human Diseases | Cancer: overview | Chemical carcinogenesis - reactive oxygen species | map05208 | 0.070175439 | 4/586 | 57/6496 | 0.7689 |
| Metabolism | Carbohydrate metabolism | Fructose and mannose metabolism | map00051 | 0.089285714 | 5/586 | 56/6496 | 0.578 |
| Metabolism | Lipid metabolism | Fatty acid biosynthesis | map00061 | 0.068965517 | 4/586 | 58/6496 | 0.7807 |
| Metabolism | Xenobiotics biodegradation and metabolism | Benzoate degradation | map00362 | 0.069444444 | 5/586 | 72/6496 | 0.7904 |
| Metabolism | Biosynthesis of other secondary metabolites | Neomycin, kanamycin and gentamicin biosynthesis | map00524 | 0.061728395 | 5/586 | 81/6496 | 0.867 |
| Metabolism | Xenobiotics biodegradation and metabolism | Styrene degradation | map00643 | 0.208333333 | 5/586 | 24/6496 | 0.05924 |
| Metabolism | Carbohydrate metabolism | Butanoate metabolism | map00650 | 0.106382979 | 5/586 | 47/6496 | 0.4201 |
| Metabolism | Energy metabolism | Carbon fixation in photosynthetic organisms | map00710 | 0.173913043 | 4/586 | 23/6496 | 0.1478 |
| Metabolism | Metabolism of terpenoids and polyketides | Monoterpenoid biosynthesis | map00902 | 0.081967213 | 5/586 | 61/6496 | 0.6551 |
| Metabolism | Biosynthesis of other secondary metabolites | Betalain biosynthesis | map00965 | 0.217391304 | 5/586 | 23/6496 | 0.05049 |
| Metabolism | Chemical structure transformation maps | Biosynthesis of terpenoids and steroids | map01062 | 0.060606061 | 6/586 | 99/6496 | 0.8932 |
| Cellular Processes | Cellular community - prokaryotes | Quorum sensing | map02024 | 0.090909091 | 5/586 | 55/6496 | 0.5615 |
| Organismal Systems | Nervous system | Synaptic vesicle cycle | map04721 | 0.416666667 | 5/586 | 12/6496 | 0.002718 |
| Organismal Systems | Endocrine system | Cortisol synthesis and secretion | map04927 | 0.333333333 | 4/586 | 12/6496 | 0.01803 |
| Human Diseases | Endocrine and metabolic disease | Cushing syndrome | map04934 | 0.307692308 | 4/586 | 13/6496 | 0.02422 |
| Human Diseases | Substance dependence | Morphine addiction | map05032 | 0.375 | 3/586 | 8/6496 | 0.02898 |
| Metabolism | Metabolism of other amino acids | Taurine and hypotaurine metabolism | map00430 | 0.25 | 6/586 | 24/6496 | 0.01726 |
| Metabolism | Lipid metabolism | Linoleic acid metabolism | map00591 | 0.214285714 | 6/586 | 28/6496 | 0.03555 |
| Metabolism | Carbohydrate metabolism | Glyoxylate and dicarboxylate metabolism | map00630 | 0.09375 | 6/586 | 64/6496 | 0.524 |
| Metabolism | Metabolism of terpenoids and polyketides | Diterpenoid biosynthesis | map00904 | 0.048387097 | 6/586 | 124/6496 | 0.9731 |
| Metabolism | Chemical structure transformation maps | Biosynthesis of alkaloids derived from terpenoid and polyketide | map01066 | 0.102040816 | 5/586 | 49/6496 | 0.4567 |
| Human Diseases | Drug resistance: antineoplastic | EGFR tyrosine kinase inhibitor resistance | map01521 | 0.5 | 1/586 | 2/6496 | 0.1723 |
| Human Diseases | Drug resistance: antineoplastic | Antifolate resistance | map01523 | 0.294117647 | 5/586 | 17/6496 | 0.01453 |
| Environmental Information Processing | Signal transduction | MAPK signaling pathway | map04010 | 0.2 | 1/586 | 5/6496 | 0.3768 |
| Environmental Information Processing | Signal transduction | ErbB signaling pathway | map04012 | 0.25 | 1/586 | 4/6496 | 0.3149 |
| Environmental Information Processing | Signal transduction | Ras signaling pathway | map04014 | 0.142857143 | 1/586 | 7/6496 | 0.4842 |
| Environmental Information Processing | Signal transduction | Rap1 signaling pathway | map04015 | 0.2 | 1/586 | 5/6496 | 0.3768 |
| Organismal Systems | Immune system | Chemokine signaling pathway | map04062 | 0.2 | 1/586 | 5/6496 | 0.3768 |
| Environmental Information Processing | Signal transduction | NF-kappa B signaling pathway | map04064 | 0.333333333 | 1/586 | 3/6496 | 0.247 |
| Environmental Information Processing | Signal transduction | Plant hormone signal transduction | map04075 | 0.5 | 6/586 | 12/6496 | 0.0003022 |
| Environmental Information Processing | Signal transduction | VEGF signaling pathway | map04370 | 0.166666667 | 1/586 | 6/6496 | 0.433 |
| Organismal Systems | Immune system | Platelet activation | map04611 | 0.071428571 | 1/586 | 14/6496 | 0.7342 |
| Organismal Systems | Immune system | Neutrophil extracellular trap formation | map04613 | 0.076923077 | 1/586 | 13/6496 | 0.7078 |
| Organismal Systems | Immune system | C-type lectin receptor signaling pathway | map04625 | 0.090909091 | 1/586 | 11/6496 | 0.6468 |
| Organismal Systems | Immune system | Natural killer cell mediated cytotoxicity | map04650 | 0.25 | 1/586 | 4/6496 | 0.3149 |
| Organismal Systems | Immune system | Th1 and Th2 cell differentiation | map04658 | 0.333333333 | 1/586 | 3/6496 | 0.247 |
| Organismal Systems | Immune system | Th17 cell differentiation | map04659 | 0.25 | 1/586 | 4/6496 | 0.3149 |
| Organismal Systems | Immune system | T cell receptor signaling pathway | map04660 | 0.25 | 1/586 | 4/6496 | 0.3149 |
| Organismal Systems | Immune system | B cell receptor signaling pathway | map04662 | 0.25 | 1/586 | 4/6496 | 0.3149 |
| Organismal Systems | Nervous system | Neurotrophin signaling pathway | map04722 | 0.2 | 1/586 | 5/6496 | 0.3768 |
| Organismal Systems | Sensory system | Phototransduction - fly | map04745 | 0.090909091 | 1/586 | 11/6496 | 0.6468 |
| Organismal Systems | Endocrine system | GnRH signaling pathway | map04912 | 0.166666667 | 1/586 | 6/6496 | 0.433 |
| Organismal Systems | Endocrine system | Estrogen signaling pathway | map04915 | 0.125 | 1/586 | 8/6496 | 0.5308 |
| Organismal Systems | Endocrine system | Thyroid hormone signaling pathway | map04919 | 0.090909091 | 1/586 | 11/6496 | 0.6468 |
| Organismal Systems | Endocrine system | Adipocytokine signaling pathway | map04920 | 0.142857143 | 1/586 | 7/6496 | 0.4842 |
| Organismal Systems | Endocrine system | Oxytocin signaling pathway | map04921 | 0.083333333 | 1/586 | 12/6496 | 0.6787 |
| Organismal Systems | Endocrine system | Renin secretion | map04924 | 0.235294118 | 4/586 | 17/6496 | 0.06053 |
| Organismal Systems | Endocrine system | Relaxin signaling pathway | map04926 | 0.166666667 | 1/586 | 6/6496 | 0.433 |
| Human Diseases | Endocrine and metabolic disease | AGE-RAGE signaling pathway in diabetic complications | map04933 | 0.111111111 | 1/586 | 9/6496 | 0.5732 |
| Organismal Systems | Endocrine system | Growth hormone synthesis, secretion and action | map04935 | 0.25 | 1/586 | 4/6496 | 0.3149 |
| Organismal Systems | Excretory system | Endocrine and other factor-regulated calcium reabsorption | map04961 | 0.125 | 1/586 | 8/6496 | 0.5308 |
| Organismal Systems | Digestive system | Fat digestion and absorption | map04975 | 0.076923077 | 1/586 | 13/6496 | 0.7078 |
| Organismal Systems | Digestive system | Vitamin digestion and absorption | map04977 | 0.153846154 | 6/586 | 39/6496 | 0.1346 |
| Human Diseases | Substance dependence | Cocaine addiction | map05030 | 0.714285714 | 5/586 | 7/6496 | 0.0001058 |
| Human Diseases | Substance dependence | Amphetamine addiction | map05031 | 0.555555556 | 5/586 | 9/6496 | 0.0005441 |
| Human Diseases | Infectious disease: bacterial | Vibrio cholerae infection | map05110 | 0.111111111 | 1/586 | 9/6496 | 0.5732 |
| Human Diseases | Infectious disease: viral | Human cytomegalovirus infection | map05163 | 0.166666667 | 1/586 | 6/6496 | 0.433 |
| Human Diseases | Infectious disease: viral | Kaposi sarcoma-associated herpesvirus infection | map05167 | 0.2 | 1/586 | 5/6496 | 0.3768 |
| Human Diseases | Infectious disease: viral | Human immunodeficiency virus 1 infection | map05170 | 0.2 | 1/586 | 5/6496 | 0.3768 |
| Human Diseases | Infectious disease: viral | Coronavirus disease - COVID-19 | map05171 | 0.1 | 1/586 | 10/6496 | 0.6117 |
| Human Diseases | Cancer: overview | Chemical carcinogenesis - DNA adducts | map05204 | 0.076923077 | 6/586 | 78/6496 | 0.7176 |
| Human Diseases | Cancer: specific types | Glioma | map05214 | 0.25 | 1/586 | 4/6496 | 0.3149 |
| Human Diseases | Cancer: specific types | Non-small cell lung cancer | map05223 | 0.166666667 | 1/586 | 6/6496 | 0.433 |
| Human Diseases | Cancer: overview | PD-L1 expression and PD-1 checkpoint pathway in cancer | map05235 | 0.25 | 1/586 | 4/6496 | 0.3149 |
| Metabolism | Carbohydrate metabolism | Galactose metabolism | map00052 | 0.152173913 | 7/586 | 46/6496 | 0.1161 |
| Metabolism | Metabolism of other amino acids | Glutathione metabolism | map00480 | 0.184210526 | 7/586 | 38/6496 | 0.05053 |
| Metabolism | Carbohydrate metabolism | Amino sugar and nucleotide sugar metabolism | map00520 | 0.050420168 | 6/586 | 119/6496 | 0.964 |
| Metabolism | Xenobiotics biodegradation and metabolism | Toluene degradation | map00623 | 0.14893617 | 7/586 | 47/6496 | 0.1265 |
| Metabolism | Metabolism of cofactors and vitamins | Riboflavin metabolism | map00740 | 0.333333333 | 8/586 | 24/6496 | 0.0008334 |
| Metabolism | Metabolism of cofactors and vitamins | Biotin metabolism | map00780 | 0.206896552 | 6/586 | 29/6496 | 0.04154 |
| Metabolism | Metabolism of cofactors and vitamins | Porphyrin metabolism | map00860 | 0.046979866 | 7/586 | 149/6496 | 0.9848 |
| Metabolism | Xenobiotics biodegradation and metabolism | Caprolactam degradation | map00930 | 0.269230769 | 7/586 | 26/6496 | 0.006733 |
| Metabolism | Biosynthesis of other secondary metabolites | Flavone and flavonol biosynthesis | map00944 | 0.137254902 | 7/586 | 51/6496 | 0.1723 |
| Metabolism | Chemical structure transformation maps | Biosynthesis of alkaloids derived from histidine and purine | map01065 | 0.166666667 | 6/586 | 36/6496 | 0.1004 |
| Metabolism | Global and overview maps | Biosynthesis of nucleotide sugars | map01250 | 0.034825871 | 7/586 | 201/6496 | 0.9995 |
| Environmental Information Processing | Signal transduction | Calcium signaling pathway | map04020 | 0.181818182 | 2/586 | 11/6496 | 0.261 |
| Environmental Information Processing | Signal transduction | HIF-1 signaling pathway | map04066 | 0.133333333 | 2/586 | 15/6496 | 0.3978 |
| Environmental Information Processing | Signal transduction | Apelin signaling pathway | map04371 | 0.222222222 | 2/586 | 9/6496 | 0.1918 |
| Organismal Systems | Immune system | Fc epsilon RI signaling pathway | map04664 | 0.181818182 | 2/586 | 11/6496 | 0.261 |
| Organismal Systems | Immune system | Fc gamma R-mediated phagocytosis | map04666 | 0.25 | 2/586 | 8/6496 | 0.1582 |
| Organismal Systems | Environmental adaptation | Circadian entrainment | map04713 | 0.222222222 | 2/586 | 9/6496 | 0.1918 |
| Organismal Systems | Environmental adaptation | Thermogenesis | map04714 | 0.086956522 | 2/586 | 23/6496 | 0.6276 |
| Organismal Systems | Nervous system | Long-term potentiation | map04720 | 0.285714286 | 2/586 | 7/6496 | 0.1259 |
| Organismal Systems | Nervous system | Retrograde endocannabinoid signaling | map04723 | 0.105263158 | 2/586 | 19/6496 | 0.5218 |
| Organismal Systems | Nervous system | Long-term depression | map04730 | 0.222222222 | 2/586 | 9/6496 | 0.1918 |
| Organismal Systems | Sensory system | Inflammatory mediator regulation of TRP channels | map04750 | 0.057142857 | 2/586 | 35/6496 | 0.8373 |
| Organismal Systems | Endocrine system | Insulin secretion | map04911 | 0.166666667 | 2/586 | 12/6496 | 0.2958 |
| Organismal Systems | Endocrine system | Melanogenesis | map04916 | 0.333333333 | 2/586 | 6/6496 | 0.09546 |
| Organismal Systems | Endocrine system | Thyroid hormone synthesis | map04918 | 0.095238095 | 2/586 | 21/6496 | 0.5771 |
| Organismal Systems | Endocrine system | GnRH secretion | map04929 | 0.222222222 | 2/586 | 9/6496 | 0.1918 |
| Organismal Systems | Digestive system | Pancreatic secretion | map04972 | 0.133333333 | 2/586 | 15/6496 | 0.3978 |
| Human Diseases | Neurodegenerative disease | Spinocerebellar ataxia | map05017 | 0.285714286 | 2/586 | 7/6496 | 0.1259 |
| Human Diseases | Infectious disease: bacterial | Shigellosis | map05131 | 0.142857143 | 2/586 | 14/6496 | 0.3644 |
| Human Diseases | Infectious disease: parasitic | African trypanosomiasis | map05143 | 0.25 | 2/586 | 8/6496 | 0.1582 |
| Human Diseases | Cancer: specific types | Hepatocellular carcinoma | map05225 | 0.142857143 | 2/586 | 14/6496 | 0.3644 |
| Metabolism | Biosynthesis of other secondary metabolites | Monobactam biosynthesis | map00261 | 0.179487179 | 7/586 | 39/6496 | 0.05701 |
| Metabolism | Lipid metabolism | Arachidonic acid metabolism | map00590 | 0.088607595 | 7/586 | 79/6496 | 0.5778 |
| Metabolism | Metabolism of cofactors and vitamins | Vitamin B6 metabolism | map00750 | 0.275862069 | 8/586 | 29/6496 | 0.003247 |
| Metabolism | Metabolism of cofactors and vitamins | Lipoic acid metabolism | map00785 | 0.159090909 | 7/586 | 44/6496 | 0.09679 |
| Metabolism | Lipid metabolism | Biosynthesis of unsaturated fatty acids | map01040 | 0.108108108 | 8/586 | 74/6496 | 0.3502 |
| Environmental Information Processing | Membrane transport | Phosphotransferase system (PTS) | map02060 | 0.122807018 | 7/586 | 57/6496 | 0.2519 |
| Environmental Information Processing | Signal transduction | Phospholipase D signaling pathway | map04072 | 0.272727273 | 3/586 | 11/6496 | 0.0697 |
| Organismal Systems | Circulatory system | Adrenergic signaling in cardiomyocytes | map04261 | 0.3 | 3/586 | 10/6496 | 0.05422 |
| Organismal Systems | Development and regeneration | Axon regeneration | map04361 | 0.428571429 | 3/586 | 7/6496 | 0.01939 |
| Organismal Systems | Nervous system | Glutamatergic synapse | map04724 | 0.375 | 3/586 | 8/6496 | 0.02898 |
| Organismal Systems | Nervous system | Cholinergic synapse | map04725 | 0.25 | 3/586 | 12/6496 | 0.08693 |
| Human Diseases | Endocrine and metabolic disease | Insulin resistance | map04931 | 0.157894737 | 3/586 | 19/6496 | 0.2424 |
| Organismal Systems | Digestive system | Salivary secretion | map04970 | 0.176470588 | 3/586 | 17/6496 | 0.1935 |
| Organismal Systems | Digestive system | Gastric acid secretion | map04971 | 0.214285714 | 3/586 | 14/6496 | 0.126 |
| Organismal Systems | Digestive system | Carbohydrate digestion and absorption | map04973 | 0.111111111 | 3/586 | 27/6496 | 0.4451 |
| Human Diseases | Substance dependence | Alcoholism | map05034 | 0.6 | 6/586 | 10/6496 | 8.04E-05 |
| Human Diseases | Cardiovascular disease | Diabetic cardiomyopathy | map05415 | 0.076923077 | 3/586 | 39/6496 | 0.6965 |
| Metabolism | Carbohydrate metabolism | Pentose and glucuronate interconversions | map00040 | 0.034482759 | 2/586 | 58/6496 | 0.9724 |
| Metabolism | Lipid metabolism | Cutin, suberine and wax biosynthesis | map00073 | 0.296296296 | 8/586 | 27/6496 | 0.001973 |
| Metabolism | Amino acid metabolism | Valine, leucine and isoleucine degradation | map00280 | 0.214285714 | 9/586 | 42/6496 | 0.01106 |
| Metabolism | Metabolism of cofactors and vitamins | Pantothenate and CoA biosynthesis | map00770 | 0.266666667 | 8/586 | 30/6496 | 0.004085 |
| Organismal Systems | Circulatory system | Vascular smooth muscle contraction | map04270 | 0.1875 | 3/586 | 16/6496 | 0.17 |
| Cellular Processes | Cellular community - eukaryotes | Gap junction | map04540 | 0.363636364 | 4/586 | 11/6496 | 0.01293 |
| Organismal Systems | Sensory system | Taste transduction | map04742 | 0.25 | 8/586 | 32/6496 | 0.006252 |
| Organismal Systems | Endocrine system | Aldosterone synthesis and secretion | map04925 | 0.136363636 | 3/586 | 22/6496 | 0.3187 |
| Organismal Systems | Endocrine system | Parathyroid hormone synthesis, secretion and action | map04928 | 0.3 | 3/586 | 10/6496 | 0.05422 |
| Human Diseases | Infectious disease: parasitic | Amoebiasis | map05146 | 0.230769231 | 3/586 | 13/6496 | 0.1057 |
| Metabolism | Lipid metabolism | Primary bile acid biosynthesis | map00120 | 0.212765957 | 10/586 | 47/6496 | 0.007974 |
| Metabolism | Amino acid metabolism | Alanine, aspartate and glutamate metabolism | map00250 | 0.357142857 | 10/586 | 28/6496 | 9.68E-05 |
| Metabolism | Amino acid metabolism | Valine, leucine and isoleucine biosynthesis | map00290 | 0.434782609 | 10/586 | 23/6496 | 1.28E-05 |
| Metabolism | Amino acid metabolism | Lysine biosynthesis | map00300 | 0.257142857 | 9/586 | 35/6496 | 0.00309 |
| Metabolism | Energy metabolism | Methane metabolism | map00680 | 0.113636364 | 10/586 | 88/6496 | 0.2683 |
| Metabolism | Biosynthesis of other secondary metabolites | Degradation of flavonoids | map00946 | 0.2 | 8/586 | 40/6496 | 0.02415 |
| Metabolism | Xenobiotics biodegradation and metabolism | Metabolism of xenobiotics by cytochrome P450 | map00980 | 0.082644628 | 10/586 | 121/6496 | 0.6623 |
| Environmental Information Processing | Signal transduction | Two-component system | map02020 | 0.169811321 | 9/586 | 53/6496 | 0.04554 |
| Environmental Information Processing | Signal transduction | Sphingolipid signaling pathway | map04071 | 0.266666667 | 4/586 | 15/6496 | 0.04005 |
| Human Diseases | Cancer: overview | Pathways in cancer | map05200 | 0.161290323 | 5/586 | 31/6496 | 0.1422 |
| Metabolism | Carbohydrate metabolism | Ascorbate and aldarate metabolism | map00053 | 0.087719298 | 5/586 | 57/6496 | 0.5941 |
| Metabolism | Metabolism of cofactors and vitamins | Ubiquinone and other terpenoid-quinone biosynthesis | map00130 | 0.154929577 | 11/586 | 71/6496 | 0.05182 |
| Metabolism | Amino acid metabolism | Arginine biosynthesis | map00220 | 0.391304348 | 9/586 | 23/6496 | 9.56E-05 |
| Metabolism | Amino acid metabolism | Cysteine and methionine metabolism | map00270 | 0.149253731 | 10/586 | 67/6496 | 0.0761 |
| Metabolism | Biosynthesis of other secondary metabolites | Biosynthesis of various antibiotics | map00998 | 0.091743119 | 10/586 | 109/6496 | 0.5268 |
| Human Diseases | Neurodegenerative disease | Pathways of neurodegeneration - multiple diseases | map05022 | 0.15625 | 5/586 | 32/6496 | 0.1568 |
| Metabolism | Amino acid metabolism | Histidine metabolism | map00340 | 0.212765957 | 10/586 | 47/6496 | 0.007974 |
| Metabolism | Metabolism of other amino acids | beta-Alanine metabolism | map00410 | 0.34375 | 11/586 | 32/6496 | 6.49E-05 |
| Metabolism | Metabolism of cofactors and vitamins | Nicotinate and nicotinamide metabolism | map00760 | 0.2 | 11/586 | 55/6496 | 0.008918 |
| Metabolism | Biosynthesis of other secondary metabolites | Flavonoid biosynthesis | map00941 | 0.162162162 | 12/586 | 74/6496 | 0.03188 |
| Metabolism | Biosynthesis of other secondary metabolites | Isoflavonoid biosynthesis | map00943 | 0.1875 | 12/586 | 64/6496 | 0.01085 |
| Metabolism | Biosynthesis of other secondary metabolites | Isoquinoline alkaloid biosynthesis | map00950 | 0.093023256 | 12/586 | 129/6496 | 0.5005 |
| Organismal Systems | Nervous system | Dopaminergic synapse | map04728 | 0.5 | 6/586 | 12/6496 | 0.0003022 |
| Organismal Systems | Endocrine system | Regulation of lipolysis in adipocytes | map04923 | 0.357142857 | 5/586 | 14/6496 | 0.0059 |
| Metabolism | Amino acid metabolism | Phenylalanine metabolism | map00360 | 0.224489796 | 11/586 | 49/6496 | 0.003561 |
| Metabolism | Lipid metabolism | alpha-Linolenic acid metabolism | map00592 | 0.272727273 | 12/586 | 44/6496 | 0.0003684 |
| Metabolism | Xenobiotics biodegradation and metabolism | Aminobenzoate degradation | map00627 | 0.162790698 | 14/586 | 86/6496 | 0.02084 |
| Metabolism | Biosynthesis of other secondary metabolites | Biosynthesis of various other secondary metabolites | map00997 | 0.147058824 | 10/586 | 68/6496 | 0.08241 |
| Organismal Systems | Digestive system | Mineral absorption | map04978 | 0.379310345 | 11/586 | 29/6496 | 2.24E-05 |
| Metabolism | Lipid metabolism | Steroid hormone biosynthesis | map00140 | 0.141414141 | 14/586 | 99/6496 | 0.05969 |
| Metabolism | Amino acid metabolism | Lysine degradation | map00310 | 0.25 | 14/586 | 56/6496 | 0.0003299 |
| Metabolism | Biosynthesis of other secondary metabolites | Biosynthesis of various alkaloids | map00996 | 0.157894737 | 12/586 | 76/6496 | 0.03827 |
| Metabolism | Chemical structure transformation maps | Biosynthesis of alkaloids derived from ornithine, lysine and nicotinic acid | map01064 | 0.161764706 | 11/586 | 68/6496 | 0.03955 |
| Metabolism | Amino acid metabolism | Phenylalanine, tyrosine and tryptophan biosynthesis | map00400 | 0.371428571 | 13/586 | 35/6496 | 5.24E-06 |
| Metabolism | Metabolism of cofactors and vitamins | Folate biosynthesis | map00790 | 0.275862069 | 16/586 | 58/6496 | 3.44E-05 |
| Metabolism | Metabolism of terpenoids and polyketides | Zeatin biosynthesis | map00908 | 0.333333333 | 13/586 | 39/6496 | 2.05E-05 |
| Metabolism | Biosynthesis of other secondary metabolites | Phenylpropanoid biosynthesis | map00940 | 0.24137931 | 14/586 | 58/6496 | 0.0004868 |
| Metabolism | Biosynthesis of other secondary metabolites | Glucosinolate biosynthesis | map00966 | 0.168831169 | 13/586 | 77/6496 | 0.01923 |
| Environmental Information Processing | Signaling molecules and interaction | Neuroactive ligand-receptor interaction | map04080 | 0.245283019 | 13/586 | 53/6496 | 0.0006522 |
| Organismal Systems | Nervous system | Serotonergic synapse | map04726 | 0.19047619 | 8/586 | 42/6496 | 0.03165 |
| Metabolism | Amino acid metabolism | Glycine, serine and threonine metabolism | map00260 | 0.291666667 | 14/586 | 48/6496 | 5.40E-05 |
| Environmental Information Processing | Signal transduction | cAMP signaling pathway | map04024 | 0.36 | 9/586 | 25/6496 | 0.0002027 |
| Human Diseases | Neurodegenerative disease | Parkinson disease | map05012 | 0.346153846 | 9/586 | 26/6496 | 0.0002856 |
| Metabolism | Nucleotide metabolism | Purine metabolism | map00230 | 0.168316832 | 17/586 | 101/6496 | 0.008351 |
| Metabolism | Metabolism of other amino acids | Cyanoamino acid metabolism | map00460 | 0.311111111 | 14/586 | 45/6496 | 2.41E-05 |
| Metabolism | Biosynthesis of other secondary metabolites | Tropane, piperidine and pyridine alkaloid biosynthesis | map00960 | 0.208333333 | 15/586 | 72/6496 | 0.001599 |
| Genetic Information Processing | Translation | Aminoacyl-tRNA biosynthesis | map00970 | 0.288461538 | 15/586 | 52/6496 | 3.39E-05 |
| Metabolism | Xenobiotics biodegradation and metabolism | Drug metabolism - cytochrome P450 | map00982 | 0.195402299 | 17/586 | 87/6496 | 0.001692 |
| Human Diseases | Cancer: overview | Central carbon metabolism in cancer | map05230 | 0.378378378 | 14/586 | 37/6496 | 1.75E-06 |
| Metabolism | Nucleotide metabolism | Pyrimidine metabolism | map00240 | 0.265625 | 17/586 | 64/6496 | 3.36E-05 |
| Metabolism | Chemical structure transformation maps | Biosynthesis of plant hormones | map01070 | 0.202898551 | 14/586 | 69/6496 | 0.002936 |
| Metabolism | Metabolism of other amino acids | D-Amino acid metabolism | map00470 | 0.260869565 | 18/586 | 69/6496 | 2.59E-05 |
| Metabolism | Amino acid metabolism | Arginine and proline metabolism | map00330 | 0.289855072 | 20/586 | 69/6496 | 1.56E-06 |
| Organismal Systems | Digestive system | Protein digestion and absorption | map04974 | 0.382978723 | 18/586 | 47/6496 | 4.57E-08 |
| Metabolism | Lipid metabolism | Glycerophospholipid metabolism | map00564 | 0.160714286 | 9/586 | 56/6496 | 0.06137 |
| Human Diseases | Cancer: overview | Choline metabolism in cancer | map05231 | 0.454545455 | 5/586 | 11/6496 | 0.001711 |
| Metabolism | Biosynthesis of other secondary metabolites | Biosynthesis of various plant secondary metabolites | map00999 | 0.140939597 | 21/586 | 149/6496 | 0.02576 |
| Metabolism | Chemical structure transformation maps | Biosynthesis of alkaloids derived from shikimate pathway | map01063 | 0.145833333 | 21/586 | 144/6496 | 0.01821 |
| Metabolism | Global and overview maps | Nucleotide metabolism | map01232 | 0.362068966 | 21/586 | 58/6496 | 1.11E-08 |
| Metabolism | Amino acid metabolism | Tyrosine metabolism | map00350 | 0.320512821 | 25/586 | 78/6496 | 8.04E-09 |
| Metabolism | Chemical structure transformation maps | Biosynthesis of phenylpropanoids | map01061 | 0.252336449 | 27/586 | 107/6496 | 5.30E-07 |
| Organismal Systems | Digestive system | Bile secretion | map04976 | 0.216494845 | 21/586 | 97/6496 | 0.0001136 |
| Metabolism | Amino acid metabolism | Tryptophan metabolism | map00380 | 0.34939759 | 29/586 | 83/6496 | 4.67E-11 |
| Environmental Information Processing | Membrane transport | ABC transporters | map02010 | 0.260869565 | 36/586 | 138/6496 | 2.49E-09 |
| Metabolism | Chemical structure transformation maps | Biosynthesis of plant secondary metabolites | map01060 | 0.281690141 | 40/586 | 142/6496 | 2.37E-11 |
| Metabolism | Global and overview maps | Biosynthesis of cofactors | map01240 | 0.170212766 | 56/586 | 329/6496 | 1.63E-06 |

Supplementary Table 10. KEGG pathway enrichment analysis

| First Category | Second Category | KEGGID | Match_status | Pathway Description | Impact_value | P_value |
| --- | --- | --- | --- | --- | --- | --- |
| Metabolism | Glycan biosynthesis and metabolism | map00552 | 2\|7 | Teichoic acid biosynthesis | 1 | 0.116012846 |
| Metabolism | Amino acid metabolism | map00250 | 10\|28 | Alanine, aspartate and glutamate metabolism | 0.832608696 | 0.00012896 |
| Metabolism | Metabolism of cofactors and vitamins | map00670 | 3\|9 | One carbon pool by folate | 0.761904762 | 0.040002472 |
| Metabolism | Lipid metabolism | map00591 | 2\|13 | Linoleic acid metabolism | 0.75 | 0.236255553 |
| Metabolism | Biosynthesis of other secondary metabolites | map00965 | 4\|11 | Betalain biosynthesis | 0.666666667 | 0.013524961 |
| Metabolism | Metabolism of cofactors and vitamins | map00750 | 7\|23 | Vitamin B6 metabolism | 0.607897153 | 0.003497419 |
| Metabolism | Amino acid metabolism | map00350 | 21\|60 | Tyrosine metabolism | 0.598215502 | 4.65E-08 |
| Metabolism | Amino acid metabolism | map00380 | 22\|56 | Tryptophan metabolism | 0.579919201 | 1.86E-09 |
| Metabolism | Metabolism of cofactors and vitamins | map00740 | 5\|21 | Riboflavin metabolism | 0.562259307 | 0.032298998 |
| Metabolism | Xenobiotics biodegradation and metabolism | map00627 | 12\|67 | Aminobenzoate degradation | 0.529372497 | 0.013494092 |
| Metabolism | Nucleotide metabolism | map00240 | 17\|62 | Pyrimidine metabolism | 0.516536853 | 3.24E-05 |
| Metabolism | Lipid metabolism | map00592 | 8\|30 | alpha-Linolenic acid metabolism | 0.507731959 | 0.004353865 |
| Metabolism | Xenobiotics biodegradation and metabolism | map00365 | 1\|8 | Furfural degradation | 0.5 | 0.37883248 |
| Metabolism | Global and overview maps | map01232 | 20\|56 | Nucleotide metabolism | 0.470250147 | 6.62E-08 |
| Metabolism | Xenobiotics biodegradation and metabolism | map00930 | 6\|18 | Caprolactam degradation | 0.468085106 | 0.004162339 |
| Metabolism | Metabolism of other amino acids | map00430 | 6\|22 | Taurine and hypotaurine metabolism | 0.400954654 | 0.011239147 |
| Metabolism | Amino acid metabolism | map00220 | 9\|23 | Arginine biosynthesis | 0.395800106 | 0.000126636 |
| Metabolism | Amino acid metabolism | map00400 | 13\|34 | Phenylalanine, tyrosine and tryptophan biosynthesis | 0.394189383 | 5.60E-06 |
| Metabolism | Lipid metabolism | map00073 | 6\|20 | Cutin, suberine and wax biosynthesis | 0.392857143 | 0.007123536 |
| Metabolism | Amino acid metabolism | map00260 | 14\|47 | Glycine, serine and threonine metabolism | 0.359733894 | 5.95E-05 |
| Metabolism | Biosynthesis of other secondary metabolites | map00943 | 11\|49 | Isoflavonoid biosynthesis | 0.345225027 | 0.003738577 |
| Metabolism | Biosynthesis of other secondary metabolites | map00940 | 14\|56 | Phenylpropanoid biosynthesis | 0.344511404 | 0.000417326 |
| Metabolism | Metabolism of cofactors and vitamins | map00770 | 7\|27 | Pantothenate and CoA biosynthesis | 0.33105939 | 0.008515312 |
| Metabolism | Amino acid metabolism | map00330 | 20\|67 | Arginine and proline metabolism | 0.325661435 | 1.68E-06 |
| Metabolism | Amino acid metabolism | map00310 | 12\|52 | Lysine degradation | 0.320744081 | 0.002047235 |
| Metabolism | Amino acid metabolism | map00340 | 8\|33 | Histidine metabolism | 0.310126582 | 0.007669071 |
| Metabolism | Amino acid metabolism | map00300 | 9\|34 | Lysine biosynthesis | 0.304758531 | 0.002737908 |
| Metabolism | Metabolism of cofactors and vitamins | map00790 | 15\|57 | Folate biosynthesis | 0.302654558 | 0.000148555 |
| Metabolism | Metabolism of cofactors and vitamins | map00760 | 10\|51 | Nicotinate and nicotinamide metabolism | 0.296338208 | 0.012902754 |
| Metabolism | Metabolism of cofactors and vitamins | map00780 | 4\|24 | Biotin metabolism | 0.28606357 | 0.118907532 |
| Genetic Information Processing | Translation | map00970 | 15\|52 | Aminoacyl-tRNA biosynthesis | 0.27027027 | 4.93E-05 |
| Metabolism | Biosynthesis of other secondary metabolites | map00996 | 3\|28 | Biosynthesis of various alkaloids | 0.265625 | 0.23282149 |
| Metabolism | Biosynthesis of other secondary metabolites | map00941 | 12\|68 | Flavonoid biosynthesis | 0.263558713 | 0.014839998 |
| Metabolism | Amino acid metabolism | map00360 | 9\|43 | Phenylalanine metabolism | 0.254188994 | 0.012060355 |
| Metabolism | Xenobiotics biodegradation and metabolism | map00624 | 3\|42 | Polycyclic aromatic hydrocarbon degradation | 0.244680851 | 0.1998137 |
| Metabolism | Amino acid metabolism | map00290 | 10\|23 | Valine, leucine and isoleucine biosynthesis | 0.242352941 | 1.84E-05 |
| Metabolism | Nucleotide metabolism | map00230 | 17\|86 | Purine metabolism | 0.237471463 | 0.001658186 |
| Metabolism | Metabolism of terpenoids and polyketides | map00903 | 2\|21 | Limonene degradation | 0.222222222 | 0.285074998 |
| Metabolism | Metabolism of terpenoids and polyketides | map00908 | 8\|26 | Zeatin biosynthesis | 0.215116279 | 0.001727806 |
| Metabolism | Amino acid metabolism | map00270 | 10\|60 | Cysteine and methionine metabolism | 0.21338459 | 0.031151659 |
| Metabolism | Biosynthesis of other secondary metabolites | map00966 | 13\|68 | Glucosinolate biosynthesis | 0.209069946 | 0.006658337 |
| Metabolism | Biosynthesis of other secondary metabolites | map00232 | 4\|18 | Caffeine metabolism | 0.206314244 | 0.06236965 |
| Metabolism | Lipid metabolism | map00140 | 14\|89 | Steroid hormone biosynthesis | 0.202229177 | 0.021114611 |
| Metabolism | Metabolism of cofactors and vitamins | map00130 | 11\|65 | Ubiquinone and other terpenoid-quinone biosynthesis | 0.199144356 | 0.02353023 |
| Metabolism | Xenobiotics biodegradation and metabolism | map00643 | 5\|24 | Styrene degradation | 0.192307692 | 0.050018895 |
| Metabolism | Biosynthesis of other secondary metabolites | map00402 | 1\|9 | Benzoxazinoid biosynthesis | 0.19047619 | 0.385488957 |
| Metabolism | Global and overview maps | map01240 | 48\|304 | Biosynthesis of cofactors | 0.182680403 | 0.000120517 |
| Metabolism | Lipid metabolism | map00564 | 7\|52 | Glycerophospholipid metabolism | 0.175853329 | 0.106249122 |
| Metabolism | Glycan biosynthesis and metabolism | map00510 | 1\|8 | N-Glycan biosynthesis | 0.166666667 | 0.37883248 |
| Metabolism | Xenobiotics biodegradation and metabolism | map00625 | 1\|31 | Chloroalkane and chloroalkene degradation | 0.163265306 | 0.145115328 |
| Metabolism | Metabolism of other amino acids | map00410 | 9\|25 | beta-Alanine metabolism | 0.153034301 | 0.000259813 |
| Metabolism | Biosynthesis of other secondary metabolites | map00901 | 3\|54 | Indole alkaloid biosynthesis | 0.148247978 | 0.128810494 |
| Metabolism | Carbohydrate metabolism | map00562 | 2\|45 | Inositol phosphate metabolism | 0.143186883 | 0.119936123 |
| Metabolism | Metabolism of other amino acids | map00470 | 16\|58 | D-Amino acid metabolism | 0.12972973 | 5.03E-05 |
| Metabolism | Biosynthesis of other secondary metabolites | map00404 | 3\|46 | Staurosporine biosynthesis | 0.125458792 | 0.176615669 |
| Metabolism | Xenobiotics biodegradation and metabolism | map00362 | 5\|67 | Benzoate degradation | 0.125164454 | 0.152532045 |
| Metabolism | Biosynthesis of other secondary metabolites | map00521 | 2\|18 | Streptomycin biosynthesis | 0.124513619 | 0.28067917 |
| Metabolism | Biosynthesis of other secondary metabolites | map00401 | 4\|29 | Novobiocin biosynthesis | 0.119531731 | 0.16115433 |
| Metabolism | Metabolism of terpenoids and polyketides | map00904 | 6\|98 | Diterpenoid biosynthesis | 0.119032178 | 0.077282835 |
| Metabolism | Carbohydrate metabolism | map00650 | 5\|44 | Butanoate metabolism | 0.109348984 | 0.172984623 |
| Metabolism | Metabolism of terpenoids and polyketides | map00902 | 4\|46 | Monoterpenoid biosynthesis | 0.108108108 | 0.201211052 |
| Metabolism | Biosynthesis of other secondary metabolites | map00960 | 6\|31 | Tropane, piperidine and pyridine alkaloid biosynthesis | 0.10806175 | 0.045259707 |
| Metabolism | Metabolism of other amino acids | map00460 | 14\|45 | Cyanoamino acid metabolism | 0.107988166 | 3.53E-05 |
| Metabolism | Energy metabolism | map00680 | 10\|83 | Methane metabolism | 0.107803313 | 0.101573168 |
| Metabolism | Lipid metabolism | map00120 | 9\|46 | Primary bile acid biosynthesis | 0.100623004 | 0.017492887 |
| Metabolism | Carbohydrate metabolism | map00051 | 5\|53 | Fructose and mannose metabolism | 0.096140787 | 0.185304669 |
| Metabolism | Xenobiotics biodegradation and metabolism | map00626 | 2\|35 | Naphthalene degradation | 0.092857143 | 0.197598647 |
| Metabolism | Carbohydrate metabolism | map00620 | 2\|28 | Pyruvate metabolism | 0.091409861 | 0.253943274 |
| Metabolism | Biosynthesis of other secondary metabolites | map00997 | 8\|53 | Biosynthesis of various other secondary metabolites | 0.089442815 | 0.066944164 |
| Metabolism | Carbohydrate metabolism | map00660 | 4\|22 | C5-Branched dibasic acid metabolism | 0.086330935 | 0.099976311 |
| Metabolism | Carbohydrate metabolism | map00630 | 6\|56 | Glyoxylate and dicarboxylate metabolism | 0.085957165 | 0.163951038 |
| Metabolism | Amino acid metabolism | map00280 | 9\|40 | Valine, leucine and isoleucine degradation | 0.084743267 | 0.007875224 |
| Metabolism | Lipid metabolism | map00565 | 2\|21 | Ether lipid metabolism | 0.081504702 | 0.285074998 |
| Metabolism | Carbohydrate metabolism | map00052 | 7\|46 | Galactose metabolism | 0.078803072 | 0.077388534 |
| Metabolism | Energy metabolism | map00720 | 4\|44 | Carbon fixation pathways in prokaryotes | 0.076441774 | 0.20464003 |
| Metabolism | Biosynthesis of other secondary metabolites | map00524 | 4\|76 | Neomycin, kanamycin and gentamicin biosynthesis | 0.07321302 | 0.076786314 |
| Metabolism | Lipid metabolism | map00561 | 3\|32 | Glycerolipid metabolism | 0.072895958 | 0.235932519 |
| Metabolism | Metabolism of terpenoids and polyketides | map01053 | 3\|17 | Biosynthesis of siderophore group nonribosomal peptides | 0.072580645 | 0.145513611 |
| Metabolism | Glycan biosynthesis and metabolism | map00540 | 1\|54 | Lipopolysaccharide biosynthesis | 0.069769847 | 0.024689101 |
| Metabolism | Xenobiotics biodegradation and metabolism | map00623 | 6\|41 | Toluene degradation | 0.063366337 | 0.101883209 |
| Metabolism | Carbohydrate metabolism | map00053 | 4\|51 | Ascorbate and aldarate metabolism | 0.061506565 | 0.186369497 |
| Metabolism | Energy metabolism | map00710 | 3\|21 | Carbon fixation in photosynthetic organisms | 0.060037523 | 0.190680049 |
| Metabolism | Metabolism of other amino acids | map00480 | 7\|38 | Glutathione metabolism | 0.05856013 | 0.040511548 |
| Metabolism | Xenobiotics biodegradation and metabolism | map00982 | 6\|55 | Drug metabolism - cytochrome P450 | 0.057971014 | 0.161819518 |
| Metabolism | Lipid metabolism | map00100 | 1\|56 | Steroid biosynthesis | 0.055090348 | 0.02090275 |
| Metabolism | Biosynthesis of other secondary metabolites | map00311 | 3\|18 | Penicillin and cephalosporin biosynthesis | 0.052173913 | 0.157984427 |
| Metabolism | Lipid metabolism | map00600 | 4\|33 | Sphingolipid metabolism | 0.051847437 | 0.185992541 |
| Metabolism | Carbohydrate metabolism | map00640 | 2\|37 | Propanoate metabolism | 0.051294657 | 0.180813162 |
| Metabolism | Carbohydrate metabolism | map00500 | 2\|35 | Starch and sucrose metabolism | 0.046616826 | 0.197598647 |
| Metabolism | Biosynthesis of other secondary metabolites | map00261 | 6\|29 | Monobactam biosynthesis | 0.04368932 | 0.035640886 |
| Metabolism | Biosynthesis of other secondary metabolites | map00950 | 9\|80 | Isoquinoline alkaloid biosynthesis | 0.043529274 | 0.124154456 |
| Metabolism | Biosynthesis of other secondary metabolites | map00944 | 4\|30 | Flavone and flavonol biosynthesis | 0.041958042 | 0.168230436 |
| Metabolism | Carbohydrate metabolism | map00020 | 2\|20 | Citrate cycle (TCA cycle) | 0.039956691 | 0.285165006 |
| Metabolism | Metabolism of terpenoids and polyketides | map00900 | 1\|43 | Terpenoid backbone biosynthesis | 0.037397863 | 0.059895583 |
| Metabolism | Metabolism of terpenoids and polyketides | map00981 | 1\|23 | Insect hormone biosynthesis | 0.035087719 | 0.241122386 |
| Metabolism | Carbohydrate metabolism | map00520 | 6\|116 | Amino sugar and nucleotide sugar metabolism | 0.034085595 | 0.035096537 |
| Metabolism | Metabolism of terpenoids and polyketides | map01055 | 2\|17 | Biosynthesis of vancomycin group antibiotics | 0.033898305 | 0.275823334 |
| Metabolism | Xenobiotics biodegradation and metabolism | map00622 | 2\|38 | Xylene degradation | 0.030204962 | 0.172560225 |
| Metabolism | Biosynthesis of other secondary metabolites | map00945 | 1\|18 | Stilbenoid, diarylheptanoid and gingerol biosynthesis | 0.026170799 | 0.312111987 |
| Metabolism | Energy metabolism | map00920 | 3\|30 | Sulfur metabolism | 0.026078235 | 0.236068677 |
| Metabolism | Xenobiotics biodegradation and metabolism | map00791 | 1\|16 | Atrazine degradation | 0.025641026 | 0.339234752 |
| Metabolism | Metabolism of cofactors and vitamins | map00860 | 5\|116 | Porphyrin metabolism | 0.024387658 | 0.017690659 |
| Metabolism | Xenobiotics biodegradation and metabolism | map00621 | 3\|36 | Dioxin degradation | 0.024087024 | 0.227234198 |
| Metabolism | Carbohydrate metabolism | map00030 | 3\|37 | Pentose phosphate pathway | 0.019444444 | 0.223634693 |
| Metabolism | Biosynthesis of other secondary metabolites | map00999 | 11\|111 | Biosynthesis of various plant secondary metabolites | 0.018249258 | 0.126284716 |
| Metabolism | Biosynthesis of other secondary metabolites | map00946 | 6\|35 | Degradation of flavonoids | 0.018115942 | 0.066953806 |
| Metabolism | Metabolism of cofactors and vitamins | map00785 | 4\|32 | Lipoic acid metabolism | 0.013242009 | 0.180673461 |
| Metabolism | Global and overview maps | map01250 | 7\|195 | Biosynthesis of nucleotide sugars | 0.012550947 | 0.000744477 |
| Metabolism | Carbohydrate metabolism | map00040 | 2\|57 | Pentose and glucuronate interconversions | 0.008178673 | 0.057476922 |
| Metabolism | Biosynthesis of other secondary metabolites | map00998 | 8\|95 | Biosynthesis of various antibiotics | 0.008152174 | 0.136594735 |
| Metabolism | Glycan biosynthesis and metabolism | map00541 | 1\|89 | O-Antigen nucleotide sugar biosynthesis | 0.006828649 | 0.001153486 |
| Metabolism | Metabolism of terpenoids and polyketides | map01057 | 1\|114 | Biosynthesis of type II polyketide products | 0.00654404 | 0.000113912 |
| Metabolism | Xenobiotics biodegradation and metabolism | map00361 | 2\|70 | Chlorocyclohexane and chlorobenzene degradation | 0.000757576 | 0.023280689 |
| Metabolism | Metabolism of terpenoids and polyketides | map00906 | 1\|102 | Carotenoid biosynthesis | 0 | 0.000349367 |
| Metabolism | Metabolism of terpenoids and polyketides | map00909 | 1\|83 | Sesquiterpenoid and triterpenoid biosynthesis | 0 | 0.001985467 |
| Metabolism | Xenobiotics biodegradation and metabolism | map00983 | 1\|39 | Drug metabolism - other enzymes | 0 | 0.081400079 |
| Metabolism | Biosynthesis of other secondary metabolites | map00332 | 4\|26 | Carbapenem biosynthesis | 0 | 0.136961454 |
| Metabolism | Lipid metabolism | map00071 | 2\|47 | Fatty acid degradation | 0 | 0.107011639 |
| Metabolism | Energy metabolism | map00910 | 2\|19 | Nitrogen metabolism | 0 | 0.283747282 |
| Metabolism | Biosynthesis of other secondary metabolites | map00331 | 1\|10 | Clavulanic acid biosynthesis | 0 | 0.387410911 |
| Metabolism | Xenobiotics biodegradation and metabolism | map00980 | 2\|68 | Metabolism of xenobiotics by cytochrome P450 | 0 | 0.026903045 |
| Metabolism | Lipid metabolism | map00062 | 1\|39 | Fatty acid elongation | 0 | 0.081400079 |
| Metabolism | Xenobiotics biodegradation and metabolism | map00984 | 1\|18 | Steroid degradation | 0 | 0.312111987 |
| Metabolism | Xenobiotics biodegradation and metabolism | map00363 | 1\|4 | Bisphenol degradation | 0 | 0.282924448 |
| Metabolism | Metabolism of other amino acids | map00440 | 1\|42 | Phosphonate and phosphinate metabolism | 0 | 0.064729017 |
| Metabolism | Metabolism of terpenoids and polyketides | map01051 | 1\|23 | Biosynthesis of ansamycins | 0 | 0.241122386 |
| Metabolism | Lipid metabolism | map01040 | 5\|36 | Biosynthesis of unsaturated fatty acids | 0 | 0.133779502 |
| Metabolism | Lipid metabolism | map00590 | 3\|46 | Arachidonic acid metabolism | 0 | 0.176615669 |
| Metabolism | Metabolism of other amino acids | map00450 | 1\|21 | Selenocompound metabolism | 0 | 0.269258757 |
| Metabolism | Metabolism of cofactors and vitamins | map00830 | 1\|17 | Retinol metabolism | 0 | 0.325959007 |
| Metabolism | Xenobiotics biodegradation and metabolism | map00642 | 1\|14 | Ethylbenzene degradation | 0 | 0.362920487 |
| Metabolism | Biosynthesis of other secondary metabolites | map00942 | 2\|41 | Anthocyanin biosynthesis | 0 | 0.148740243 |
| Metabolism | Carbohydrate metabolism | map00010 | 1\|31 | Glycolysis / Gluconeogenesis | 0 | 0.145115328 |
| Metabolism | Biosynthesis of other secondary metabolites | map00333 | 1\|18 | Prodigiosin biosynthesis | 0 | 0.312111987 |
| Metabolism | Metabolism of terpenoids and polyketides | map01059 | 1\|55 | Biosynthesis of enediyne antibiotics | 0 | 0.022721194 |
| Metabolism | Metabolism of cofactors and vitamins | map00730 | 1\|29 | Thiamine metabolism | 0 | 0.166091878 |
| Metabolism | Metabolism of terpenoids and polyketides | map00907 | 3\|33 | Pinene, camphor and geraniol degradation | 0 | 0.234726766 |
| Metabolism | Lipid metabolism | map00061 | 4\|57 | Fatty acid biosynthesis | 0 | 0.161010404 |

Supplementary Table 11. KEGG pathway topology analysis

| Metabolite | Escherichia-Shigella | Candidatus_Arthromitus | Pantoea | Lactococcus | Mycoplasma | Fusobacterium | Bartonella | unclassified_f__Christensenellaceae | norank_f__Muribaculaceae | unclassified_f__Enterobacteriaceae | unclassified_o__Lactobacillales | Myroides | Sphingobacterium | Rhodococcus | Vagococcus | Enterobacter | Serratia | Acinetobacter | Enterococcus | unclassified_f__Synergistaceae |
| --- | --- | --- | --- | --- | --- | --- | --- | --- | --- | --- | --- | --- | --- | --- | --- | --- | --- | --- | --- | --- |
| (9Z)-12-Hydroxyhexadecenoylcarnitine | 0.03739 | 0.18812 | 0.28476 | 0.391 | 0.62384 | 0.87289 | 0.391 | 0.49254 | 0.62384 | 0.62384 | 0.28476 | 0.74706 | 0.93471 | 1 | 0.71769 | 0.62384 | 0.18812 | 0.28476 | 0.10409 | 0.11761 |
| 8-Hydroxytrtradeca-10,12-dienoylcarnitine | 0.391 | 0.87289 | 0.74706 | 0.18812 | 0.391 | 0.62384 | 0.62384 | 0.49254 | 0.391 | 0.87289 | 0.74706 | 0.28476 | 0.32172 | 0.50463 | 0.45018 | 0.391 | 0.03739 | 0 | 0.28476 | 0.11761 |
| Decanoylcarnitine | 0.391 | 0.87289 | 0.74706 | 0.18812 | 0.391 | 0.62384 | 0.62384 | 0.49254 | 0.391 | 0.87289 | 0.74706 | 0.28476 | 0.32172 | 0.50463 | 0.45018 | 0.391 | 0.03739 | 0 | 0.28476 | 0.11761 |
| Isovalerylcarnitine | 0.391 | 0.74706 | 0.87289 | 0.03739 | 0.391 | 0.28476 | 0.28476 | 0.32172 | 0.391 | 0.10409 | 0.87289 | 0.18812 | 0.55281 | 0.62384 | 0.71769 | 1 | 0.10409 | 0.391 | 0.74706 | 0.45018 |
| O-Acetylcarnitine | 0.10409 | 0.391 | 0.62384 | 0.10409 | 0.28476 | 0.391 | 0.18812 | 0.21889 | 0.28476 | 0.391 | 0.62384 | 0.391 | 0.74058 | 0.87289 | 0.71769 | 0.87289 | 0.03739 | 0.18812 | 0.28476 | 0.11761 |
| 1-Methylcyclohexane-1,2-dicarboxylic acid | 0.10409 | 0.391 | 0.62384 | 0.10409 | 0.28476 | 0.391 | 0.18812 | 0.21889 | 0.28476 | 0.391 | 0.62384 | 0.391 | 0.74058 | 0.87289 | 0.71769 | 0.87289 | 0.03739 | 0.18812 | 0.28476 | 0.11761 |
| Dioctyl succinate | 1 | 0.87289 | 0.62384 | 0.74706 | 1 | 0.62384 | 0.62384 | 0.80483 | 1 | 0.03739 | 0.62384 | 0.87289 | 0.74058 | 0.87289 | 0.21517 | 0.391 | 0.87289 | 0.391 | 0.50463 | 0.58109 |
| Trans-Cinnamic acid | 0.62384 | 0.87289 | 0.62384 | 0.74706 | 0.28476 | 0.62384 | 0.62384 | 0.43377 | 0.28476 | 0.28476 | 0.62384 | 0.87289 | 0.8696 | 0.87289 | 0.58109 | 0.87289 | 0.391 | 0.18812 | 0.391 | 0.21517 |
| Norleucine | 0.62384 | 0.87289 | 0.62384 | 0.74706 | 0.28476 | 0.62384 | 0.62384 | 0.43377 | 0.28476 | 0.28476 | 0.62384 | 0.87289 | 0.8696 | 0.87289 | 0.58109 | 0.87289 | 0.391 | 0.18812 | 0.391 | 0.21517 |
| Eplerenone | 0.18812 | 0.391 | 0.18812 | 0.74706 | 0.50463 | 1 | 0.62384 | 0.55281 | 0.50463 | 0.391 | 0.74706 | 1 | 0.93471 | 0.87289 | 0.71769 | 0.50463 | 0.28476 | 0.10409 | 0.03739 | 0.04052 |
| 1-(4-ethoxyphenyl)-3-[2-(3-methylphenyl)imidazo[1,2-a]pyridin-3-yl]urea | 0.18812 | 0.391 | 0.18812 | 0.74706 | 0.50463 | 1 | 0.62384 | 0.55281 | 0.50463 | 0.391 | 0.74706 | 1 | 0.93471 | 0.87289 | 0.71769 | 0.50463 | 0.28476 | 0.10409 | 0.03739 | 0.04052 |
| 4-Pentenoic acid | 0.50463 | 0.62384 | 0.28476 | 0.87289 | 0.62384 | 0.87289 | 0.87289 | 0.74058 | 0.62384 | 0.10409 | 1 | 0.74706 | 0.93471 | 0.74706 | 0.58109 | 0.62384 | 0.62384 | 0.28476 | 0.18812 | 0.21517 |
| Cyclohexane | 0.50463 | 0.62384 | 0.28476 | 0.87289 | 0.62384 | 0.87289 | 0.87289 | 0.74058 | 0.62384 | 0.10409 | 1 | 0.74706 | 0.93471 | 0.74706 | 0.58109 | 0.62384 | 0.62384 | 0.28476 | 0.18812 | 0.21517 |
| Tetradecanoylcarnitine | 0.391 | 0.62384 | 0.87289 | 0.28476 | 0 | 0.03739 | 0.03739 | 0.00482 | 0 | 0.74706 | 0.87289 | 0.62384 | 0.8696 | 0.62384 | 0.58109 | 0.391 | 0.18812 | 0.391 | 0.62384 | 0.21517 |
| Hydroxytetradecadienyl-l-carnitine | 0.87289 | 0.74706 | 0.50463 | 0.50463 | 0.10409 | 0.03739 | 0.28476 | 0.1718 | 0.10409 | 0.74706 | 0.391 | 0.62384 | 0.93471 | 0.74706 | 0.71769 | 0.18812 | 0.62384 | 0.87289 | 0.62384 | 0.85794 |
| 6-Hydroxyhexadecanoylcarnitine | 0.74706 | 1 | 0.62384 | 0.18812 | 0.03739 | 0 | 0.10409 | 0.05385 | 0.03739 | 0.391 | 0.62384 | 0.391 | 0.93471 | 0.87289 | 0.58109 | 0.28476 | 0.28476 | 0.62384 | 0.87289 | 0.58109 |
| (12E)-10-Hydroxytetradec-12-enoylcarnitine | 0.28476 | 0.62384 | 0.50463 | 0.391 | 0.18812 | 0.50463 | 0.391 | 0.269 | 0.18812 | 0.62384 | 0.87289 | 0.62384 | 0.74058 | 1 | 0.71769 | 0.74706 | 0.10409 | 0.03739 | 0.18812 | 0.04052 |
| Pc(18:0/0:0) | 0.28476 | 0.62384 | 0.391 | 0.50463 | 0.74706 | 0.87289 | 0.87289 | 0.80483 | 0.74706 | 0.62384 | 0.87289 | 0.62384 | 0.49254 | 0.62384 | 0.45018 | 0.18812 | 0.18812 | 0.03739 | 0.10409 | 0.11761 |
| Choline | 0 | 0.03739 | 0.10409 | 0.62384 | 0.391 | 0.74706 | 0.18812 | 0.269 | 0.391 | 0.87289 | 0.18812 | 0.87289 | 0.61438 | 0.50463 | 0.45018 | 1 | 0.28476 | 0.391 | 0.03739 | 0.04052 |
| Neryl rhamnosyl-glucoside | 0 | 0.03739 | 0.10409 | 0.62384 | 0.391 | 0.74706 | 0.18812 | 0.269 | 0.391 | 0.87289 | 0.18812 | 0.87289 | 0.61438 | 0.50463 | 0.45018 | 1 | 0.28476 | 0.391 | 0.03739 | 0.04052 |
| Taurocholic Acid | 0 | 0.03739 | 0.10409 | 0.62384 | 0.391 | 0.74706 | 0.18812 | 0.269 | 0.391 | 0.87289 | 0.18812 | 0.87289 | 0.61438 | 0.50463 | 0.45018 | 1 | 0.28476 | 0.391 | 0.03739 | 0.04052 |
| 13'-Carboxy-alpha-tocotrienol | 0.18812 | 0.391 | 0.18812 | 0.74706 | 0.50463 | 1 | 0.62384 | 0.55281 | 0.50463 | 0.391 | 0.74706 | 1 | 0.93471 | 0.87289 | 0.71769 | 0.50463 | 0.28476 | 0.10409 | 0.03739 | 0.04052 |
| N-Linoleoyl Histidine | 0.03739 | 0.10409 | 0.03739 | 0.87289 | 0.62384 | 0.87289 | 0.50463 | 0.55281 | 0.62384 | 0.62384 | 0.28476 | 0.74706 | 0.74058 | 0.62384 | 0.85794 | 0.62384 | 0.391 | 0.28476 | 0 | 0.04052 |
| LysoPC(18:1(11Z)/0:0) | 1 | 0.74706 | 0.87289 | 0.87289 | 0.391 | 0.28476 | 0.28476 | 0.32172 | 0.391 | 0.62384 | 0.87289 | 0.62384 | 0.21889 | 0.18812 | 0.11761 | 0 | 0.74706 | 0.391 | 0.62384 | 0.85794 |
| Saponin A | 0.87289 | 0.62384 | 0.87289 | 1 | 0.74706 | 0.50463 | 0.391 | 0.55281 | 0.74706 | 0.18812 | 0.50463 | 0.74706 | 0.32172 | 0.391 | 0.04052 | 0.10409 | 0.74706 | 0.28476 | 0.62384 | 0.71769 |
| Ketamine | 0.391 | 0.28476 | 0.18812 | 0.62384 | 0.391 | 0.18812 | 0.74706 | 0.55281 | 0.391 | 0.87289 | 0.10409 | 0.50463 | 0.74058 | 0.87289 | 0.85794 | 0.391 | 0.87289 | 1 | 0.28476 | 0.71769 |
| 11'-Carboxy-gamma-chromanol | 0.391 | 0.28476 | 0.18812 | 0.62384 | 0.391 | 0.18812 | 0.74706 | 0.55281 | 0.391 | 0.87289 | 0.10409 | 0.50463 | 0.74058 | 0.87289 | 0.85794 | 0.391 | 0.87289 | 1 | 0.28476 | 0.71769 |
| Palmitoylcarnitine | 0.391 | 0.28476 | 0.18812 | 0.62384 | 0.391 | 0.18812 | 0.74706 | 0.55281 | 0.391 | 0.87289 | 0.10409 | 0.50463 | 0.74058 | 0.87289 | 0.85794 | 0.391 | 0.87289 | 1 | 0.28476 | 0.71769 |
| 13-Hydroxyoctadecanoylcarnitine | 0.87289 | 0.391 | 0.28476 | 0.03739 | 0.62384 | 0.391 | 0.87289 | 0.74058 | 0.62384 | 0.391 | 0.28476 | 0 | 0.05385 | 0.10409 | 0.45018 | 0.62384 | 0.18812 | 0.28476 | 0.74706 | 0.85794 |
| L-Tyrosine | 0.87289 | 0.62384 | 0.391 | 0.28476 | 0.74706 | 0.87289 | 0.87289 | 0.80483 | 0.74706 | 0.10409 | 0.87289 | 0.18812 | 0.269 | 0.18812 | 0.85794 | 0.62384 | 0.62384 | 0.87289 | 0.62384 | 0.71769 |

Supplementary Table 12. Correlation analysis
